# A serial multiparametric quantitative magnetic resonance imaging study to assess proteoglycan depletion of human articular cartilage and its effects on functionality

**DOI:** 10.1038/s41598-020-72208-y

**Published:** 2020-09-15

**Authors:** Tobias Hafner, Justus Schock, Manuel Post, Daniel Benjamin Abrar, Philipp Sewerin, Kevin Linka, Matthias Knobe, Christiane Kuhl, Daniel Truhn, Sven Nebelung

**Affiliations:** 1 Department of Diagnostic and Interventional Radiology, Aachen University Hospital, Aachen, Germany; 2 Medical Faculty, Department of Diagnostic and Interventional Radiology, University Hospital Düsseldorf, Moorenstraße 5, 40225 Dusseldorf, Germany; 3 Institute of Computer Vision and Imaging, RWTH University Aachen, Aachen, Germany; 4 Medical Faculty, Department and Hiller-Research-Unit for Rheumatology, University Hospital Düsseldorf, Düsseldorf, Germany; 5 Department of Continuum and Materials Mechanics, Hamburg University of Technology, Hamburg, Germany; 6 Clinic for Orthopaedic and Trauma Surgery, Cantonal Hospital Luzern, Luzern, Switzerland

**Keywords:** Medical research, Experimental models of disease, Biomaterials, Musculoskeletal system, Functional magnetic resonance imaging, Magnetic resonance imaging

## Abstract

Water, collagen, and proteoglycans determine articular cartilage functionality. If altered, susceptibility to premature degeneration is increased. This study investigated the effects of enzymatic proteoglycan depletion on cartilage functionality as assessed by advanced Magnetic Resonance Imaging (MRI) techniques under standardized loading. Lateral femoral condylar cartilage-bone samples from patients undergoing knee replacement (n = 29) were serially imaged by Proton Density-weighted and T1, T1ρ, T2, and T2* mapping sequences on a clinical 3.0 T MRI scanner (Achieva, Philips). Using pressure-controlled indentation loading, samples were imaged unloaded and quasi-statically loaded to 15.1 N and 28.6 N, and both before and after exposure to low-concentrated (LT, 0.1 mg/mL, n = 10) or high-concentrated trypsin (HT, 1.0 mg/mL, n = 10). Controls were not treated (n = 9). Responses to loading were assessed for the entire sample and regionally, i.e. sub- and peri-pistonally, and zonally, i.e. upper and lower sample halves. Trypsin effects were quantified as relative changes (Δ), analysed using appropriate statistical tests, and referenced histologically. Histological proteoglycan depletion was reflected by significant sub-pistonal decreases in T1 (*p* = 0.003) and T2 (*p* = 0.008) after HT exposure. Loading-induced changes in T1ρ and T2* were not related. In conclusion, proteoglycan depletion alters cartilage functionality and may be assessed using serial T1 and T2 mapping under loading.

## Introduction

Cartilage degeneration is the hallmark change of osteoarthritis (OA), a potentially devastating and permanently debilitating condition that affects hundreds of millions of people globally, i.e. 3.3–3.6% of the world’s population. In the United States, radiographic evidence of OA is present in approximately 80% of the senior population, i.e. > 65 years of age, even though only 60% of these individuals have symptoms^1^. By and large, these findings have been confirmed for Germany, too^2^. Beyond the considerable impact on individual patients, OA has considerable societal and economic impact, which led to its declaration as a "priority disease" by the World Health Organisation^3^. Its medical and economic burden is projected to increase further as the principal risk factors, i.e. age and obesity, are projected to rise in incidence^4^.

Pathomechanistically, cartilage degeneration is the result of abnormal tissue remodelling secondary to predisposing tissue susceptibility, unfavourable biomechanical conditions, and persistent inflammation^5^. On a structural and compositional level, the tissue undergoes progressive changes of its key constituents, i.e. collagen and proteoglycans, that comprise the extracellular matrix (ECM). During early degeneration, proteoglycan content is lost, while the collagen network is increasingly disoriented and disrupted, which leads to increased water uptake, tissue swelling, and softening^6^. If progressive, the degenerative changes result in advanced tissue disintegration and, eventually, tissue loss.

In consideration of the deficits of clinical-standard morphological Magnetic Resonance Imaging (MRI) techniques in the detection of early, potentially reversible, cartilage degeneration^7–9^, quantitative MRI (qMRI) techniques and alternative biophysical methodologies such as solid-state nuclear magnetic resonance (NMR) have been studied in clinical and research contexts to assess the tissue beyond mere morphology^10^. While solid-state NMR spectroscopy provides atomic-level insight into the structure, dynamics, and functionality of cartilage and bone^11–14^, qMRI techniques spatially map the tissue’s distinct biophysical properties such as T2 or T1ρ relaxation characteristics in a pixel-wise manner, thereby providing markers of (ultra)structure and/or composition. Recently, qMRI techniques have been combined with mechanical loading to study the tissue in more functional contexts and beyond the unloaded configuration. For additional details, the reader is referred to related comprehensive review articles^15,16^. The rationale behind these approaches is to assess the tissue in more functional contexts by mapping its imaging features in both the unloaded and loaded configurations to determine changes as surrogate parameters of functionality. In a variety of *in-vitro*, *in-situ*, and *in-vivo* studies, physiological and pathological responses to loading could be discerned as promising indicators of the cartilage status in health and disease^17–22^. Hence, a solid body of scientific and clinical evidence indicates that aberrant response-to-loading patterns are associated with altered tissue integrity and functionality. Nonetheless, the interrelatedness of the tissue’s structural and compositional properties and resultant functionality remains to be fully understood as it determines the joint’s susceptibility to OA and OA progression.

Therefore, the objective of this study was to systematically investigate the role of proteoglycan depletion in load bearing of human articular cartilage. To this end, 20 human articular cartilage samples were exposed to trypsin in two different concentrations to simulate one of the earliest changes in cartilage degeneration and OA^6^ in a well-established pathomimetic model^23^. Nine samples served as controls. The samples’ response to loading as a marker of tissue functionality was assessed before and after incubation without (control study arm) or with trypsin (trypsin exposure study arms) using serial T1, T1ρ, T2, and T2* mapping under standardized indentation loading in three different configurations, i.e. unloaded, moderate loading, and strong loading. Based on this imaging-based basic research framework, we aimed to bring together compositional changes induced by controlled *in-vitro* degradation of cartilage and the imaging correlates of physiological and pathological functionality as assessed by the relative changes under loading of T1, T1ρ, T2, and T2*. Our hypotheses are i) that trypsin exposure alters cartilage composition and cartilage functionality as a function of concentration and ii) that T1, T1ρ, T2, and T2* are reflective of these alterations and thereby provided imaging features of physiological and pathological functionality.

## Results

All 29 cartilage samples completed evaluation by qMRI and histology.

**Figure 1:**
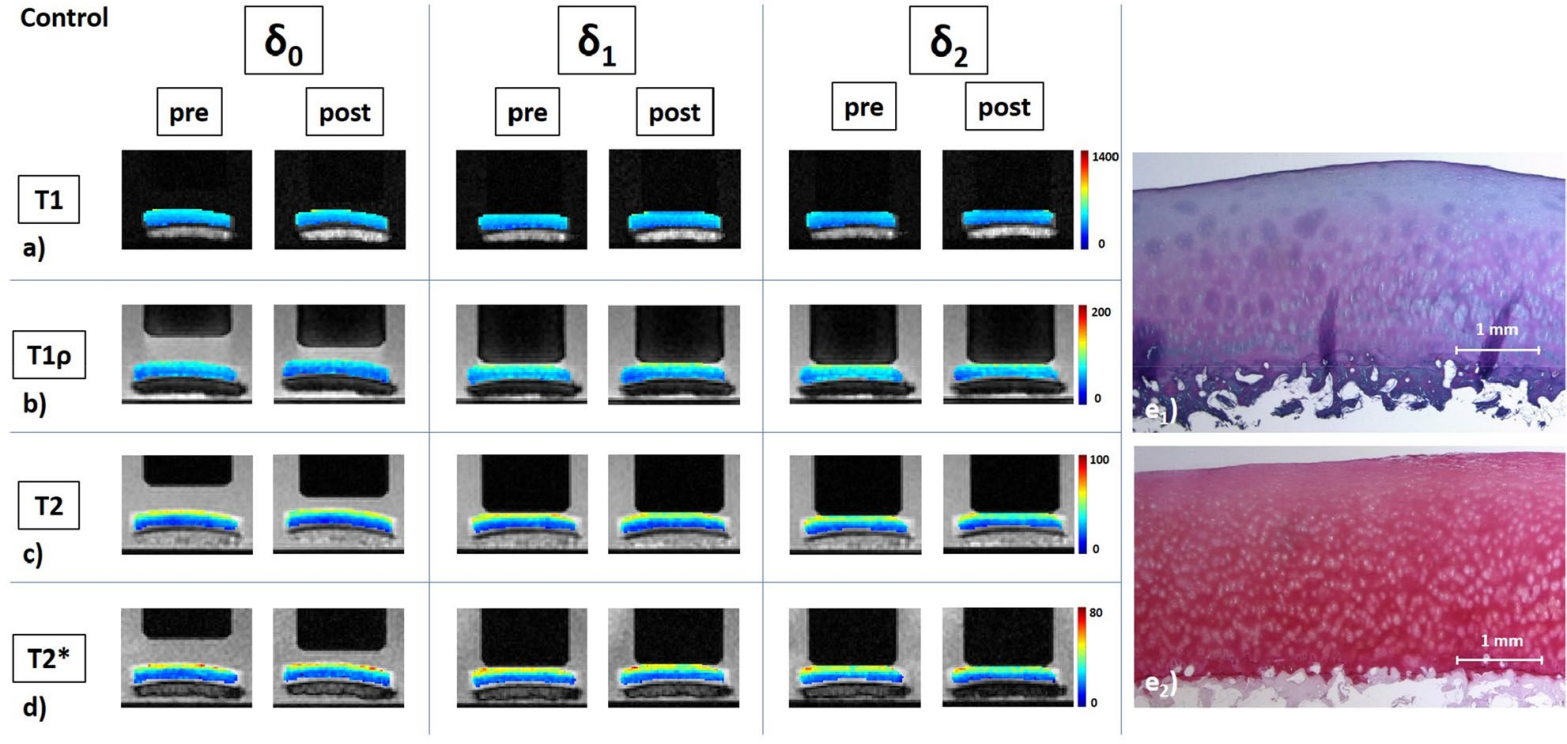
Details of a control cartilage sample and its response to loading as assessed by serial quantitative MR images as a function of loading intensity. Serially acquired T1 (**a**), T1ρ (**b**), T2 (**c**), and T2* (**d**) maps of a representative control sample before (pre) and after (post) subjection to incubation under standardized conditions (37 °C, 5% CO_2_, and 21% O_2_) without additional trypsin exposure. Loading intensity was controlled by set pressure levels and measurements were made in the unloaded configuration (δ_0_) and under loading to 15.1 N (δ_1_) and 28.6 N (δ_2_). Segmented color-coded relaxation maps were overlaid onto the corresponding morphological images. Unit of color codes on the right is [ms]. Of note, the loading piston’s diameter is 10 mm. Corresponding histological sections of the cartilage sample after Hematoxylin and eosin (**e**_**1**_) and Safranin O (**e**_**2**_) staining indicate structural and compositional integrity of the sample. More specifically, the tissue surface and tidemark were intact, while cellularity and Safranin-O staining intensity were regular.

**Figure 2:**
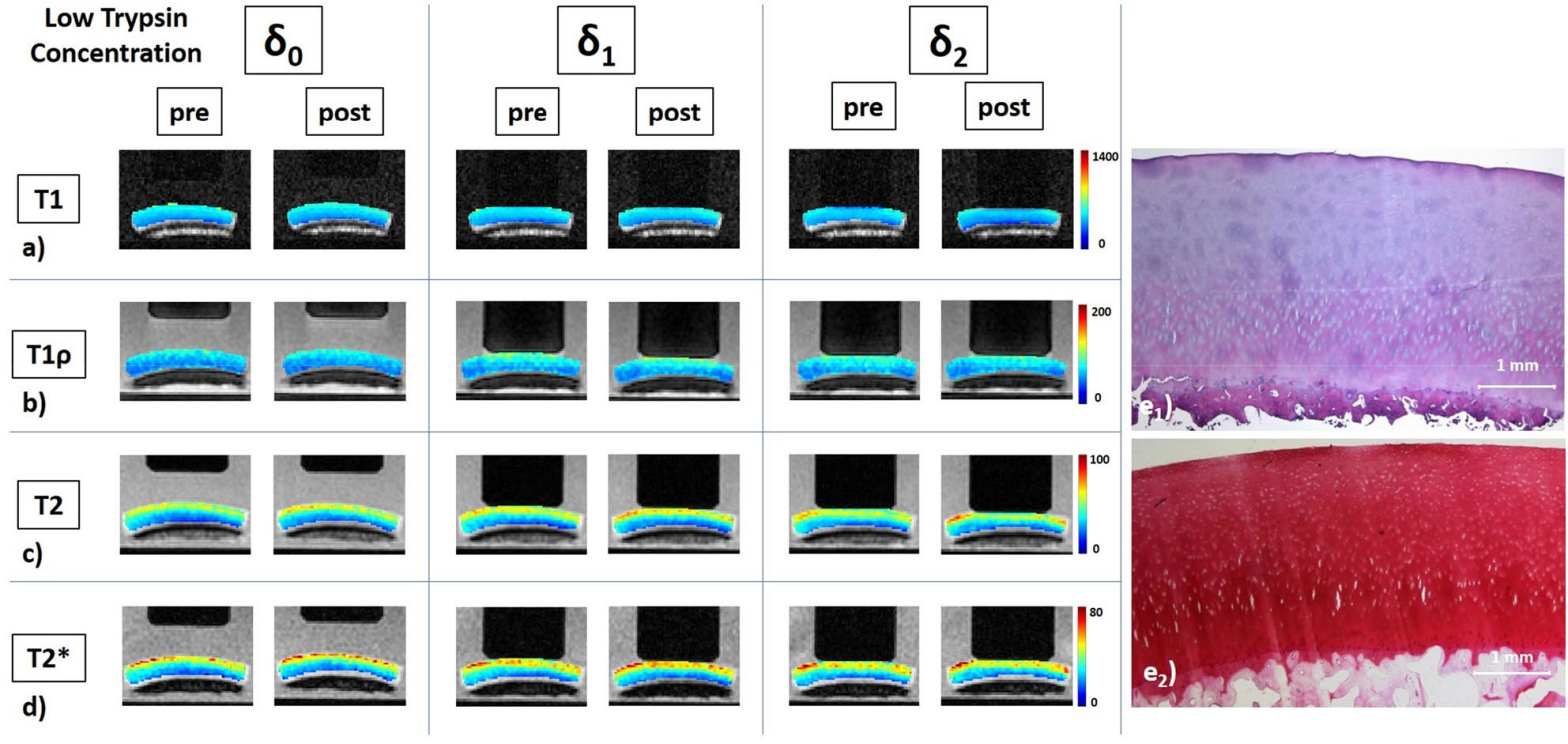
Details of a low-concentration trypsin-treated cartilage sample, its response to loading, and corresponding histological sections. Serial quantitative T1 (**a**), T1ρ (**b**), T2 (**c**), and T2* (**d**) maps as well as corresponding histological sections (e) of a representative sample before and after exposure to low concentration of trypsin at 0.1 mg/mL for 2 h. Histological assessment revealed intact tissue surface and tidemark as well as regular cellularity (**e**_**1**_), but superficial Safranin-O de-staining indicative of proteoglycan depletion that was limited to the superficial tissue zone (**e**_**2**_). Otherwise, image details as in Fig. 1.

**Figure 3:**
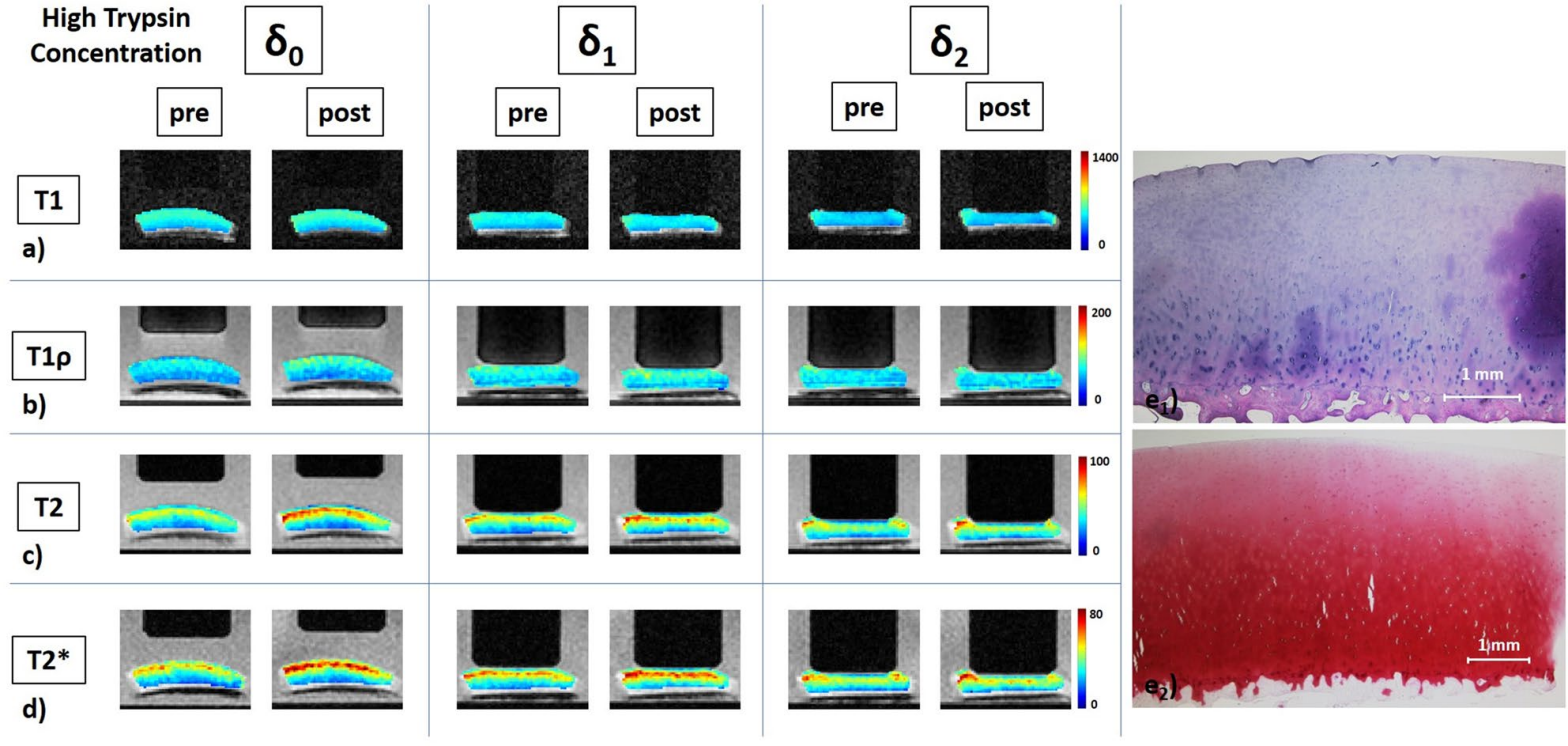
Details of a high-concentration trypsin-treated cartilage sample, its response to loading, and corresponding histological sections. Serial quantitative T1 (**a**), T1ρ (**b**), T2 (**c**), and T2* (**d**) maps as well as corresponding histological sections (**e**) of a representative sample before and after exposure to high concentration of trypsin at 1.0 mg/mL for 2 h. Histological assessment revealed intact tissue surface and tidemark as well as regular cellularity (**e**_**1**_), but expansive Safranin-O de-staining indicative of advanced proteoglycan depletion of the superficial und transitional tissue zones (**e**_**2**_). Superficial artefacts secondary to histological preparation. Otherwise, image details as in Fig. 1.

Histological evaluation of cartilage tissue adjacent to the cartilage sample revealed gross tissue integrity throughout as signified by mean Mankin sum scores of 1.9 ± 1.1 (range, 0–4), equalling Mankin Grade (MG) 0 in all tissues. Of note, most adjacent tissues had Mankin sum scores of 1 or 2, i.e. slight pre-existent signs of degeneration such as focal hypercellularity, surface fibrillation or Safranin-O de-staining. After incubation without trypsin exposure control samples did not demonstrate any structural or compositional changes on post-exposure evaluation, as indicated by intact tissue surface, regular cellularity, and no Safranin-O de-staining (Fig. 1). After exposure to low concentration of trypsin (LT), samples exhibited superficial Safranin-O de-staining indicative of slight proteoglycan depletion that was limited to the superficial tissue zone (Fig. 2). After exposure to high concentration of trypsin (HT), however, Safranin-O de-staining was progressive and involved the superficial and transitional tissue zones. No surface disintegration, cellular changes or extracellular (ECM) disruption were noted (Fig. 3).

In the following, δ_0_ refers to the absolute qMRI parameter values in the unloaded reference configuration, while δ_1_ and δ_2_ refer to the respective values under loading of 15.1 N (δ_1_) and 28.6 N (δ_2_). Consequently, relative changes in the values at δ_1_ or δ_2_ versus δ_0_ are referred to as Δ_1_ or Δ_2_.

In response to loading, T1, T1ρ, T2, and T2* underwent variable zonal and regional changes as a function of trypsin exposure and loading intensity. Table 1 outlines the relative changes of the unloaded reference configuration post-exposure (δ_0post_) as compared to the unloaded reference configuration pre-exposure (δ_0pre_). Table 2 outlines the relative changes of the different loading magnitudes, i.e. δ_1_ and δ_2_, as compared to the unloaded configuration δ_0_, both before and after trypsin exposure. Supplementary Table S3 details the absolute qMRI parameter values at δ_0_, δ_1_, and δ_2_.

**Table 1:** Details of Δ_0_, i.e. relative changes of T1, T1ρ, T2, and T2* at δ_0_ during the pre- and post-exposure measurement series, as a function of trypsin exposure and region-of-interest.

	Δ_0_	*p* value
**T1**		
*Low trypsin*		
Entire	0.4 ± 2.9	0.846
SPA	− 0.4 ± 3.2	0.625
SPA upper	− 0.1 ± 1.8	0.922
SPA lower	− 0.7 ± 6.5	0.625
PPA	1.4 ± 2.8	0.232
PPA upper	2.1 ± 2.3	0.027
PPA lower	0.7 ± 5.0	0.695
*High trypsin*		
Entire	1.9 ± 3.5	0.131
SPA	1.9 ± 3.8	0.131
SPA upper	4.0 ± 3.3	**0.004**
SPA lower	− 0.4 ± 6.1	0.846
PPA	2.1 ± 3.5	0.193
PPA upper	4.6 ± 3.2	**0.004**
PPA lower	− 2.6 ± 5.2	0.275
*Controls*		
Entire	− 1.3 ± 1.3	0.027
SPA	− 0.9 ± 1.6	0.084
SPA upper	− 1.2 ± 2.0	0.105
SPA lower	− 1.1 ± 2.5	0.275
PPA	− 1.3 ± 2.2	0.131
PPA upper	− 0.8 ± 2.3	0.275
PPA lower	− 1.4 ± 3.7	0.275
**T1ρ**		
*Low trypsin*		
Entire	0.6 ± 7.9	0.922
SPA	0.4 ± 7.5	0.999
SPA upper	0.6 ± 8.6	0.999
SPA lower	0.2 ± 8.4	0.770
PPA	− 0.7 ± 9.7	0.999
PPA upper	0.5 ± 11.4	0.922
PPA lower	− 1.6 ± 9.9	0.999
*High trypsin*		
Entire	3.3 ± 11.9	0.492
SPA	4.2 ± 13.4	0.322
SPA upper	5.6 ± 15.5	0.322
SPA lower	2.9 ± 12.6	0.557
PPA	1.4 ± 12.6	0.846
PPA upper	4.1 ± 12.3	0.557
PPA lower	− 1.8 ± 14.5	0.770
*Controls*		
Entire	− 12.1 ± 8.1	**0.002**
SPA	− 12.2 ± 7.7	**0.002**
SPA upper	− 11.8 ± 9.2	**0.002**
SPA lower	− 12.7 ± 9.4	**0.004**
PPA	− 12.3 ± 9.3	**0.004**
PPA upper	− 12.1 ± 8.8	**0.004**
PPA lower	− 12.8 ± 11.2	**0.004**
**T2**		
*Low trypsin*		
Entire	4.5 ± 9.7	0.232
SPA	4.3 ± 10.2	0.275
SPA upper	5.6 ± 11.1	0.193
SPA lower	2.5 ± 10.5	0.846
PPA	4.7 ± 9.5	0.105
PPA upper	6.0 ± 11.4	0.105
PPA lower	4.1 ± 10.6	0.432
*High trypsin*		
Entire	9.5 ± 10.7	0.020
SPA	10.1 ± 10.2	0.014
SPA upper	10.9 ± 11.1	0.014
SPA lower	8.7 ± 12.1	0.049
PPA	9.5 ± 13.4	0.064
PPA upper	10.6 ± 13.4	0.049
PPA lower	8.5 ± 15.9	0.193
*Controls*		
Entire	− 12.4 ± 8.3	**0.002**
SPA	− 12.5 ± 9.1	**0.002**
SPA upper	− 11.2 ± 6.0	**0.002**
SPA lower	− 14.6 ± 13.6	**0.004**
PPA	− 12.1 ± 6.3	**0.004**
PPA upper	− 9.9 ± 5.6	**0.004**
PPA lower	− 16.4 ± 9.7	**0.004**
T2*		
*Low trypsin*		
Entire	5.8 ± 8.9	0.105
SPA	5.8 ± 8.8	0.105
SPA upper	6.5 ± 10.1	0.105
SPA lower	4.7 ± 12.2	0.375
PPA	5.3 ± 9.3	0.131
PPA upper	6.3 ± 10.9	0.160
PPA lower	4.9 ± 12.7	0.557
*High trypsin*		
Entire	7.4 ± 8.5	0.014
SPA	8.5 ± 8.6	**0.010**
SPA upper	9.7 ± 9.3	0.020
SPA lower	8.5 ± 10.6	0.027
PPA	7.3 ± 12.9	0.275
PPA upper	7.5 ± 12.0	0.160
PPA lower	8.4 ± 19.6	0.432
*Controls*		
Entire	− 8.3 ± 9.1	0.014
SPA	− 8.5 ± 10.7	0.037
SPA upper	− 7.8 ± 7.5	**0.010**
SPA lower	− 9.8 ± 16.1	0.105
PPA	− 6.5 ± 9.4	0.049
PPA upper	− 6.3 ± 9.2	0.049
PPA lower	− 8.2 ± 15.6	0.049

Δ_0_ was calculated as Δ_0_ = ((δ_0post_/δ_0pre_) − 1) * 100 [%], where δ_0post_ and δ_0pre_ are the respective post- and pre-exposure absolute parameter values at δ_0_. Data are mean ± standard deviation [%]. Wilcoxon matched pairs test was used to assess differences between δ_0pre_ and δ_0post_. Significant differences are indicated in bold type.

**Table 2:** Details of Δ_1_ and Δ_2_, i.e. relative changes of T1, T1ρ, T2, and T2* at δ_1_ versus δ_0_ and at δ_2_ versus δ_0_ during the pre- and post-exposure measurement series, as a function of trypsin exposure, region-of-interest, and loading intensity.

	Pre exposure		Post exposure		*p* value	
	Δ_1pre_	Δ_2pre_	Δ_1post_	Δ_2post_	Δ_1pre_ vs. Δ_1post_	Δ_2pre_ vs. Δ_2post_
**T1**						
*Low trypsin*						
Entire	− 3.7 ± 2.3	− 7.4 ± 3.2	− 3.4 ± 2.4	− 7.1 ± 2.6	1.000	1.000
SPA	− 6.0 ± 2.2	− 10.2 ± 3.3	− 5.0 ± 2.7	− 10.4 ± 3.8	1.000	1.000
SPA upper	− 8.6 ± 2.8	− 15.5 ± 4.6	− 8.0 ± 3.2	− 15.4 ± 4.1	0.922	0.922
SPA lower	− 2.6 ± 3.7	− 3.5 ± 4.2	− 0.9 ± 4.1	− 3.8 ± 5.1	0.584	0.880
PPA	− 0.6 ± 2.7	− 3.5 ± 3.3	− 1.0 ± 3.0	− 3.1 ± 3.0	1.000	1.000
PPA upper	− 0.9 ± 2.8	− 4.7 ± 3.6	− 1.8 ± 4	− 4.3 ± 3.8	0.566	0.568
PPA lower	0.2 ± 4.7	− 1.5 ± 5.0	0.4 ± 2.9	− 0.5 ± 6.2	1.000	1.000
*High trypsin*						
Entire	− 5.0 ± 3.5	− 10.4 ± 4.2	− 6.4 ± 3.5	− 11.5 ± 2.4	0.720	0.720
SPA	− 6.3 ± 4.1	− 12.6 ± 5.0	− 9.0 ± 2.6	− 15.2 ± 2.7	0.106	0.092
SPA upper	− 9.6 ± 5.1	− 17.2 ± 6.5	− 15.1 ± 5.4	− 23.0 ± 5.8	**0.002**	**0.003**
SPA lower	− 2.0 ± 4.2	− 6.7 ± 4.6	− 0.8 ± 6.8	− 4.6 ± 4.1	0.706	0.494
PPA	− 2.7 ± 3.3	− 6.7 ± 4.0	− 2.4 ± 5.2	− 6.2 ± 3.6	1.000	1.000
PPA upper	− 4.0 ± 4.8	− 8.4 ± 5.6	− 4.8 ± 5.2	− 9.0 ± 5.1	1.000	1.000
PPA lower	− 1.7 ± 4.0	− 4.3 ± 2.6	2.4 ± 8.8	− 0.2 ± 6.1	0.172	0.152
*Controls*						
Entire	− 4.3 ± 2.5	− 8.4 ± 2.1	− 4.2 ± 2.2	− 8.1 ± 3.1	1.000	1.000
SPA	− 4.9 ± 2.4	− 9.2 ± 2.3	− 5.5 ± 3.1	− 9.6 ± 3.2	0.650	0.745
SPA upper	− 7.7 ± 4.5	− 13.9 ± 4.1	− 8.8 ± 4.0	− 14.8 ± 3.3	0.594	0.594
SPA lower	− 2.1 ± 2.8	− 4.1 ± 3.6	− 1.7 ± 3.7	− 2.6 ± 5.0	0.908	0.908
PPA	− 2.8 ± 2.9	− 6.7 ± 4.4	− 2.1 ± 1.8	− 5.9 ± 4.0	0.768	0.768
PPA upper	− 3.9 ± 3.1	− 8.7 ± 5.0	− 3.5 ± 2.7	− 9.0 ± 5.1	1.000	1.000
PPA lower	− 0.9 ± 3.6	− 2.1 ± 4.7	0.2 ± 4.0	− 1.2 ± 5.3	1.000	1.000
**T1ρ**						
*Low trypsin*						
Entire	13.3 ± 3.6	15.5 ± 6.2	12.2 ± 4.5	12.0 ± 7.4	0.367	0.140
SPA	15.5 ± 4.5	16.9 ± 7.9	13.8 ± 6.0	13.2 ± 7.2	1.000	1.000
SPA upper	21.3 ± 6.4	20.9 ± 7.9	18.8 ± 5.9	16.7 ± 8.3	0.324	0.324
SPA lower	9.4 ± 8.1	12.8 ± 11.3	8.7 ± 11.8	10.3 ± 12.5	0.765	0.082
PPA	9.1 ± 4.1	12.6 ± 4.6	9.9 ± 6.0	11.3 ± 10.0	1.000	1.000
PPA upper	12.6 ± 6	15.8 ± 5.2	13.6 ± 9.4	14.3 ± 11.4	1.000	1.000
PPA lower	5.4 ± 5.7	9.1 ± 6.1	5.5 ± 10.1	7.9 ± 13.1	1.000	1.000
*High trypsin*						
Entire	13.5 ± 8.2	15.4 ± 9.4	12.6 ± 10.2	17.1 ± 26.2	1.000	1.000
SPA	16.1 ± 9.2	17.6 ± 10.7	14.4 ± 12.1	20.2 ± 32.9	1.000	1.000
SPA upper	18.2 ± 9.5	17.3 ± 13.1	15.0 ± 13.5	17.1 ± 30.8	1.000	1.000
SPA lower	13.8 ± 9.8	18.4 ± 10.5	14.0 ± 13.4	24.6 ± 36.5	1.000	1.000
PPA	8.2 ± 8.4	11.4 ± 9.3	8.3 ± 9.3	10.7 ± 9.3	1.000	1.000
PPA upper	9.1 ± 8.7	11.6 ± 8.0	7.2 ± 9.8	7.4 ± 12.0	0.508	0.486
PPA lower	6.9 ± 9.7	11.1 ± 11.8	10.1 ± 11.8	15.8 ± 11.8	0.646	0.646
*Controls*						
Entire	8.9 ± 4.6	9.3 ± 5.7	13.3 ± 6.1	15.9 ± 7.0	0.084	0.034
SPA	10.8 ± 4.4	11.5 ± 5.6	15.3 ± 5.4	17.8 ± 6.6	0.061	0.040
SPA upper	14.8 ± 6.3	13.4 ± 6.2	19.3 ± 8.5	20.8 ± 9.4	0.104	0.070
SPA lower	6.7 ± 9.3	9.6 ± 10.7	11.4 ± 11.2	14.9 ± 12.2	0.187	0.166
PPA	4.3 ± 6.6	4.2 ± 8.1	8.2 ± 7.7	11.4 ± 8.7	0.209	0.030
PPA upper	5.2 ± 7.5	5.3 ± 7.7	11.5 ± 7.8	12.9 ± 9.6	0.059	0.012
PPA lower	3.2 ± 10.3	2.8 ± 11.5	4.9 ± 9.1	9.7 ± 9.4	0.665	0.200
**T2**						
*Low trypsin*						
entire	5.5 ± 3.2	4.2 ± 5.6	2.4 ± 3.8	1.9 ± 7.2	0.064	0.237
SPA	5.0 ± 4.4	2.9 ± 7.7	1.4 ± 3.9	− 0.2 ± 7.6	0.244	0.667
SPA upper	5.1 ± 5.7	1.4 ± 7.5	0.0 ± 5.5	− 4.3 ± 8.8	0.020	0.076
SPA lower	4.9 ± 5.9	5.9 ± 12.2	3.6 ± 6.0	6.3 ± 8.7	1.000	1.000
PPA	6.2 ± 3.3	6.4 ± 4.9	4.1 ± 4.6	5.6 ± 9.1	0.244	0.667
PPA upper	8.0 ± 2.7	7.1 ± 5.1	4.9 ± 5.0	5.7 ± 9.3	0.042	0.466
PPA lower	4.2 ± 5.6	6.2 ± 6.1	2.1 ± 6.3	5.1 ± 10.8	0.842	0.842
*High trypsin*						
Entire	1.1 ± 3.3	− 0.3 ± 5.1	− 1.5 ± 4.2	− 6.2 ± 6.4	0.210	0.026
SPA	− 0.2 ± 4.7	− 2.9 ± 7.4	− 3.0 ± 6.2	− 9.9 ± 9.0	0.235	**0.008**
SPA upper	− 0.5 ± 6.5	− 7.4 ± 9.9	− 4.9 ± 8.4	− 15.2 ± 11.8	0.188	0.014
SPA lower	0.2 ± 4.7	3.7 ± 7.1	0.2 ± 7.7	− 0.9 ± 10.8	0.998	0.126
PPA	3.5 ± 5.4	3.8 ± 5.1	0.9 ± 5.5	1.0 ± 6.8	0.468	0.468
PPA upper	4.7 ± 6.1	3.8 ± 7.0	1.3 ± 6.5	0.0 ± 7.8	0.328	0.328
PPA lower	1.7 ± 7.2	3.0 ± 6.1	0.2 ± 6.3	3.1 ± 7.4	1.000	1.000
*Controls*						
Entire	0.2 ± 3.8	− 2.9 ± 4.7	− 1.1 ± 4.7	− 2.8 ± 4.7	0.986	0.997
SPA	− 0.5 ± 4.4	− 3.7 ± 5.2	− 2.1 ± 5.0	− 4.4 ± 4.5	0.892	0.892
SPA upper	0.8 ± 3.3	− 5.8 ± 4	− 1.4 ± 5.5	− 5.9 ± 4.9	0.366	0.961
SPA lower	− 1.6 ± 6.1	− 0.9 ± 8.1	− 3.2 ± 5.6	− 2.0 ± 8.9	1.000	1.000
PPA	2.4 ± 4.6	− 0.2 ± 5.7	1.2 ± 5.0	0.5 ± 7.2	1.000	1.000
PPA upper	2.9 ± 5.1	− 0.5 ± 5.2	3.6 ± 3.6	0.2 ± 7.9	1.000	1.000
PPA lower	0.5 ± 5.9	− 1.1 ± 6.4	0.3 ± 8.1	1.4 ± 8.5	0.926	0.470
T2*						
*Low trypsin*						
Entire	8.3 ± 6.8	7.0 ± 6.1	5.0 ± 3.9	3.2 ± 4.9	0.136	0.136
SPA	8.2 ± 7.7	6.1 ± 8.4	3.9 ± 4.3	1.5 ± 5.1	0.310	0.310
SPA upper	5.1 ± 8.7	2.5 ± 8.6	− 0.1 ± 7.1	− 2.6 ± 7.0	0.028	0.061
SPA lower	12.7 ± 11	11.9 ± 11.4	10.7 ± 9.5	8.8 ± 6.0	0.868	0.868
PPA	8.4 ± 6.2	8.7 ± 4.5	6.6 ± 4.3	6.3 ± 7.6	0.310	0.310
PPA upper	8.6 ± 6.4	7.7 ± 4.8	5.4 ± 7.4	4.9 ± 8.8	**0.002**	0.253
PPA lower	8.8 ± 6.6	11.2 ± 5.7	7.9 ± 7.4	8.0 ± 10.0	0.806	0.806
*High trypsin*						
Entire	3.6 ± 4.3	1.2 ± 6.1	2.7 ± 6.3	− 8.2 ± 14.6	0.657	0.046
SPA	2.2 ± 5.4	− 1.5 ± 8.1	1.6 ± 8.8	− 11.6 ± 17.2	0.800	0.074
SPA upper	0.3 ± 7.4	− 6.8 ± 10.3	− 2.9 ± 11.5	− 16.3 ± 18.4	0.327	0.056
SPA lower	5.3 ± 6.4	7.4 ± 9.7	7.2 ± 8.2	− 4.1 ± 20.2	0.248	0.130
PPA	6.2 ± 5.3	6.2 ± 5.5	4.7 ± 6.3	− 0.8 ± 13.1	0.463	0.088
PPA upper	4.7 ± 6.8	4.5 ± 9.1	2.2 ± 9.6	− 1.3 ± 11.8	0.365	0.046
PPA lower	8.9 ± 8.7	8.3 ± 7.2	8.4 ± 7.7	1.0 ± 18.3	0.875	0.500
*Controls*						
Entire	2.0 ± 5.3	− 0.5 ± 6.1	0.1 ± 5.2	− 2.3 ± 4.5	0.788	0.788
SPA	1.0 ± 5.8	− 1.3 ± 6.5	− 1.2 ± 5.6	− 3.9 ± 4.5	0.552	0.552
SPA upper	0.8 ± 5.5	− 3.3 ± 6.4	− 2.7 ± 5.6	− 7.2 ± 4.5	0.230	0.230
SPA lower	1.9 ± 7.2	1.1 ± 9.6	1.1 ± 6.8	0.8 ± 7.3	1.000	1.000
PPA	6.4 ± 10.6	3.0 ± 8.8	2.5 ± 4.6	0.9 ± 7.0	0.730	0.730
PPA upper	5.6 ± 10.2	0.8 ± 9.1	3.8 ± 5.3	0.2 ± 8.1	1.000	1.000
PPA lower	7.5 ± 20.3	6.2 ± 14.6	3.7 ± 8.4	3.2 ± 9.4	1.000	1.000

Pre- and post-exposure values of Δ_1_ and Δ_2_ were comparatively evaluated using paired Student's *t*-tests. Data are mean ± standard deviation [%]. Significant differences are indicated in bold type.

Generally, we observed substantial variability in response to loading in terms of absolute values and relative changes as indicated by high measures of statistical spread such as standard deviations and interquartile ranges throughout the study.

By trend, **T1** at δ_0_ was consistently decreased in controls after incubation without enzyme exposure. After LT exposure, however, changes were variable with negligible decreases in the sub-pistonal area (SPA) and slight increases in the peri-pistonal area (PPA). In contrast, after HT exposure, we observed moderate, yet significant increases in the entire samples’ upper halves, both sub-pistonally (SPA_upper_ [HT], Δ_0_ = 4.0 ± 3.3%, *p* = 0.004) and peri-pistonally (PPA_upper_ [HT], Δ_0_ = 4.6 ± 3.2%, *p* = 0.004) as compared to slight and non-significant decreases in the lower halves (Table 1). Under loading, changes in T1 were dominated by decreases and -by and large- associated with strain, i.e. Δ_1_ < Δ_2_, particularly for the SPA and the upper halves. For the PPA, results differed with similar strain-related decreases in T1 in the upper sample halves (PPA_upper_) and ambiguous changes in the lower sample halves (PPA_lower_). In all samples, regardless of trypsin exposure, loading-induced changes in T1 demonstrated the following pattern of characteristic changes: Relative changes in the SPA were more pronounced than in the PPA and relative changes in the upper halves more pronounced than in the lower halves. Of note, these patterns were found for T1ρ and T2, too. Consequently, SPA_upper_ and PPA_lower_ displayed largest and smallest loading-induced decreases in T1, respectively. Overall, no significant differences between pre- and post-exposure relative changes in T1 were found in controls and after LT exposure. However, decreases in T1 were significantly larger after HT exposure in the SPA_upper_, both in response to moderate (SPA_upper_ [LT], Δ_1pre_ = − 9.6 ± 5.1%; Δ_1post_ = − 15.1 ± 5.4%; *p* = 0.002) and strong loading (SPA_upper_ [LT], Δ_2pre_ = − 17.2 ± 6.5%; Δ_2post_ = − 23.0 ± 5.8%; *p* = 0.003). In other regions-of-interest (ROIs), decreases in T1 were not significantly different (Table 2).

**T1ρ** at δ_0_ was consistently and significantly decreased in controls after incubation without enzyme exposure, e.g. (entire cartilage sample (ECS) [controls], Δ_0_ = − 12.1 ± 8.1%, p = 0.002), while hardly any changes were found after LT and HT exposure (Table 1). Even though dominated by considerable increases throughout, the T1ρ-associated responses to loading were characterized by substantial statistical variability. Consequently, we did not find any significant pre- vs. post-exposure differences in the loading-induced changes of T1ρ, regardless of trypsin exposure. If at all present, the association of relative changes with strain was only weak. While in controls, increases in T1ρ tended to be greater after incubation, no such trends were observed after LT and HT exposure (Table 2).

**T2** at δ_0_ was significantly decreased after incubation without enzyme exposure in all ROIs of controls, similar to T1ρ (ECS [controls], Δ_0_ = − 12.4 ± 8.3%, *p* = 0.002), while after LT and HT exposure, T2 at δ_0_ was non-significantly increased (Table 1). Overall, T2 tended to decrease sub-pistonally and to increase peri-pistonally. While sub-pistonal changes were associated with strain, i.e. relative changes under moderate loading were smaller than under strong loading (Δ_1_ < Δ_2_), that was not the case for the largely ambiguous peri-pistonal changes. No significant differences in the pre- vs. post exposure loading responses were found in controls or after LT exposure, while after HT exposure, sub-pistonal decreases were significantly larger (SPA [HT], Δ_2pre_ = − 2.9 ± 7.4%; Δ_2post_ = − 9.9 ± 9.0%; *p* = 0.008). The significant sub-pistonal decreases were primarily driven by changes in the upper sample halves (Table 2).

Like T1ρ and T2, T2* at δ_0_ was substantially decreased in controls after incubation without enzyme exposure. Decreases in T2* at δ_0_ reached or tended towards significance in all ROIs of controls, e.g. (SPA_upper_ [controls], Δ_0_ = − 7.8 ± 7.5%, *p* = 0.01). In contrast, T2* at δ_0_ was substantially increased after LT and HT exposure, e.g. (SPA [HT], Δ_0_ = 8.5 ± 8.6%, *p* = 0.01) (Table 1). Loading-induced changes in T2* were ambiguous without a clear pattern and seemingly not related to loading intensity. Even though characterized by large standard deviations, T2* tended to decrease sub-pistonally and to increase peri-pistonally. Significant pre- and post-exposure differences were only determined for the PPA_lower_ after LT exposure (PPA_lower_ [LT], Δ_1pre_ = 8.6 ± 6.4%; Δ_1post_ = 5.4 ± 7.4%; *p* = 0.002), while controls and HT exposed samples did not demonstrate significant differences. After HT exposure, we observed divergent increases in T2* under moderate loading alongside decreases under strong loading (Table 2).

Absolute values of T1, T1ρ, T2, and T2* (Supplementary Table S3) and T1, T1ρ, T2, and T2* maps (Figs. 1, 2 and 3, Supplementary Figure S1) were largely reflective of the above-mentioned changes. In controls (Fig. 1), no gross changes were noted after incubation without enzyme exposure. Pre- and post-exposure samples displayed characteristic decreases in the qMRI parameters as a function of tissue depth. Similarly, the response-to-loading patterns were not largely different with largest changes observed sub-pistonally. Peri-pistonal and deep tissue areas displayed least loading-induced changes.

After LT exposure (Fig. 2), slight changes were seen at δ_0_ that primarily involved the superficial tissue zone and sub-pistonal region, particularly in the T2 and T2* maps. Loading-induced changes tended to be more intense and widespread. In keeping with the pattern outlined above, deep and peri-pistonal areas underwent less changes than superficial and sub-pistonal areas.

After HT exposure (Fig. 3), the depth-wise stratification of the qMRI parameters became a lot clearer, particularly in the T2 and T2* maps. The tissue underwent substantial morphological deformation and flattening with increasing loading. Largest changes were noted for T1, where homogeneous decreases were observed sub-pistonally, and for T2 and T2*, where superficial increases were more marked. In other samples (Supplementary Figure S1), changes were considerably less pronounced. Increases of superficial zones at δ_0_ were accompanied by more intense and widespread loading-induced changes.

Cartilage sample heights at δ_0_, i.e. in the unloaded reference configuration, were slightly reduced in controls (from 2.77 ± 0.19 mm [pre exposure] to 2.66 ± 0.22 mm [post exposure], *p* = 0.016) and after LT exposure (from 2.59 ± 0.32 mm [pre] to 2.55 ± 0.31 mm [post], *p* = 0.373), while they were marginally increased after HT exposure (from 2.40 ± 0.41 mm [pre] to 2.43 ± 0.41 mm [post], *p* = 0.343). Additionally, serial measurements of sample heights at δ_0_, δ_1_, and δ_2_ indicated significant decreases in response to loading, irrespective of trypsin exposure (*p* < 0.001) (Supplementary Table S1). Correspondingly, pixel numbers, i.e. the number of pixels contained within the (sub-)segmentation outlines, were reflective of these changes. In response to loading, pixel numbers decreased significantly in all directly loaded regions-of-interest (ROIs), i.e. the sub-pistonal area (SPA), and in the upper sample halves of the peri-pistonal area (PPA_upper_), though significant only after trypsin exposure. Otherwise, not directly loaded regions, i.e. the entire peri-pistonal area (PPA), did not demonstrate significant pre- and post-exposure differences in pixel numbers (Supplementary Table S2).

## Discussion

The most important findings of this study are that (1) proteoglycan depletion secondary to trypsin exposure affects human articular cartilage functionality and its imaging features and (2) the altered response-to-loading patterns demonstrate regional and zonal variability and may be best assessed using T2 and, to a lesser extent, T1 mapping.

In this pathomimetic in vitro model, trypsin was selected to induce depletion of proteoglycans and non-collagenous macromolecules because a solid body of scientific evidence details its effects on articular cartilage, thereby allowing comparative analyses^24,25^. As a serine protease, trypsin cleaves peptide bonds of proteoglycans and brings about their selective degradation as a function of concentration and exposure duration. Notably, trypsin-induced proteoglycan depletion is largely inconsistent and affected by the initial proteoglycan distribution and concentration, location within the joint, cartilage thickness, and surrounding medium. In bovine retropatellar cartilage, Moody et al. determined approximately 46% and 71% proteoglycan loss following exposure to 0.1 and 1.0 mg/mL trypsin for 2 h^23^. The trypsin concentrations in the present study were informed by these data and confirmed by other studies that used Safranin-O staining for reference purposes and demonstrated proteoglycan loss of 76% after exposure to 2.5 mg/mL trypsin over 3 h^26^ and of 51% after exposure to 0.1 mg/mL trypsin for 2 h^27^. Despite these studies’ differences in terms of species, trypsin type, and cartilage location, topography, and integrity, these studies are largely in line with one another and with our findings. Histologically, we found slight-to-moderate Safranin O de-staining of the superficial and transitional zones, confirming effective proteoglycan depletion as a function of concentration. Yet, at this point we can only speculate whether trypsin-induced degradation is specific (and exclusive) to proteoglycans. In contrast to the commonly held belief that -apart from specific collagenases- collagen fibrils are resistant to enzymatic cleavage^28,29^, previous studies have provided anecdotical evidence that human trypsin-2 is indeed capable of cleaving collagen-type II in human cartilage, while animal trypsin is not^30^. Consequently, in the absence of advanced biochemical reference methodology to prove or disprove trypsin specificity, the following discussion is based on the premise that porcine trypsin (as used in this study) did not affect other ECM constituents.

During physiological loading, intact articular cartilage is subjected to water redistribution, both within the tissue from loaded to less loaded areas and into and out of the tissue. Concomitantly, the ECM experiences condensation and deformation, relative increases in proteoglycan and collagen contents, and collagen fibre re-orientation^31–33^. These response-to-loading mechanisms may be reflected by the imaging characteristics of pre-exposure samples. For once, sample height decreased with loading. For another, the qMRI parameters underwent distinct changes. In this context, it is important to note that -with the exception of dGEMRIC (delayed gadolinium-enhanced MRI of cartilage) no qMRI parameter has exclusive specificity to any particular cartilage constituent. **T1** decreased considerably and as a function of loading intensity with most pronounced decreases in the sub-pistonal upper sample halves. This is plausible in light of this marker’s primary predisposition to intra-tissue water^34^, resulting in longer T1 relaxation with higher fluid fractions^35^, while collagen or proteoglycans seem to contribute less^36^. **T1ρ**, however, increased under loading though not clearly related to strain. Interpretation is not straightforward as this parameter’s specificity profile remains unclear with water, collagen and proteoglycan content as well as collagen fibre orientation assumed to contribute to T1ρ characteristics^37,38^. Under loading, the deeper radial collagen fibers display fiber crimping^31,39^, which increases the percentage of fibers oriented at magic angle and reduces residual dipolar interaction, thereby increasing T1ρ (as well as T2) values. Moreover, recent literature reports have disproven the previously assumed proteoglycan specificity of T1ρ and qualified this association as weak^35,40,41^ so that loading-induced compositional changes may only be secondary. Consequently, T1ρ nowadays is considered a marker of the tissue’s macromolecular constitution with sensitivity to its solid and fluid constituents. Recently, positive correlations of T1ρ with fluid fraction and collagen fiber orientation, and negative correlations with collagen and proteoglycan content were reported^35^. Moreover, earlier studies by us and others have reported this parameter’s exquisite mechanosensitivity and loading-induced changes in T1ρ in a range of pre-clinical and clinical contexts^17,21,22,42,43^. **T2** underwent divergent changes in response to loading, i.e. sub-pistonal decreases and peri-pistonal increases. While the former was associated with strain, the latter was not. As T2 is widely considered a marker of collagen fibre orientation and density, collagen content and network organisation, and intra-tissue water^37,44^, its discrepant changes most likely reflect the complex interplay of these constituents during loading and are largely in line with earlier literature data^45,46^. However, the relation between T2 and proteoglycan content remains unclear. Studying suspensions of chondroitin sulphate in experimental contexts, which is the most abundant sulphated glycosaminoglycan of cartilage proteoglycans^47^, Menezes et al. found an exponential association with higher T2 values indicating lower proteoglycan concentrations^37^. In the same study, a similar association was determined for T2 and collagen concentration. In light of the constitution of the ECM which is primarily made up of collagens (70%) and proteoglycans (20%)^47^, collagen is dominating -by far- over proteoglycan in both constituents’ contributions to T2 relaxation. These findings were later confirmed by other studies^48,49^. T2* was subject to ambiguous changes without a clear pattern or any obvious relation to strain. Susceptible to the magnetic susceptibility effects of inherently inhomogeneous cartilage, T2* supposedly reflects collagen microstructure rather than collagen content^50,51^. Like T2, T2* is sensitive towards water and its interactions with collagen, while its association with proteoglycans is still debated^50^. Earlier reports have reported an inverse relation, i.e. higher T2* values indicating lower proteoglycan and collagen contents^35^. However, the ambiguity of loading-induced changes in T2* does not allow solid conclusions to be made.

Substantial increases in T1, T2, and T2* were after HT exposure, mostly driven by changes in the upper sample halves. This is not surprising as proteoglycan depletion causes an influx of water^52^ and these three qMRI parameters are closely associated with hydration^35,53^. Moreover, the lower proteoglycan concentration itself probably contributes to these increase^35,48^. Further, the above-mentioned response-to-loading patterns were altered. **First**, significantly larger decreases in T1 and T2 were found after HT exposure, but only in the SPA_upper_ (T1) or SPA (T2), respectively. It is important to note that the significant differences in T2 were not present in the unloaded configuration, thereby highlighting the potential value of cartilage functionality assessment and rendering T2 particularly suitable for assessing altered functionality after proteoglycan depletion. Otherwise, pre- and post-exposure changes in T1, T2 or T2* were not significantly different. This finding is plausible as, physiologically, proteoglycans pre-stress the collagen network by osmotic pressure^54^. Proteoglycan depletion, however, removes this pre-stress, thereby reducing mechanical properties, which renders the tissue more compliant and viscous, and subsequently facilitates water flow ^29^. These are the most likely imaging correlate of the pronounced decreases in T1 and T2. Therefore, proteoglycan depletion -one of the earliest signs of cartilage degeneration and OA^6^—compromises the load-bearing capacity of the superficial tissue (and surface), thereby promoting susceptibility to further damage to other tissue regions. Notably, the mechanical effects of trypsin are far more widespread than suggested by histological Safranin O de-staining. Griffin et al. determined trypsin-induced histological proteoglycan loss to a depth of up to 200 μm, while mechanical effects were noted to a depth of 500 μm^29^. Even when supplemented by more advanced reference measures such as optical absorbance measurements, proteoglycan concentration is variably affected by trypsin^23^, so that absolute quantification of proteoglycan content, let alone assessment of its integrity and functionality, may be challenging to realize.

**Second**, trypsin supposedly left the underlying collagen network’s microstructural architecture intact. Beyond histological referencing, this is evidenced by the fact that, overall, T2* and T1ρ, both parameters with sensitivity towards functional collagen properties^35,44^, were otherwise not significantly altered after trypsin exposure. Herein, it is worth realizing that T1ρ was not significantly affected by proteoglycan depletion, which is surprising, given this parameter’s sensitivity towards intra-tissue changes as detailed above. Most likely, these observations are due to the overpowering dominance of collagen as compared to proteoglycans in both constituents’ contribution to T1ρ relaxation^37^. Hence, proteoglycan depletion of the superficial and upper transitional tissue zones, i.e. those zones that display the lowest proteoglycan content anyway^55^, seems largely irrelevant to T1ρ characteristics, lending more evidence to the theory that proteoglycan content and T1ρ are largely unrelated.

Pre-exposure measurements under loading and incubation without enzyme exposure had significant effects on T1ρ, T2, and T2* that underwent significant decreases in controls. Probably, these decreases are related to the tissue’s stress relaxation after loading and an indication of ongoing ultrastructural recovery. The rate of stress relaxation is associated with strain and proceeds significantly slower after higher strains^56^. Consequently, full recovery may necessitate hours to days^57^ and may not have been completed. This, of course, limits repeatability and may be only partially remedied by intra-sample referencing. Incomplete ultrastructural recovery could also be a reason for the substantial standard deviations observed throughout the study. Regardless of the exact underlying cause, the tissue’s loading and cultivation history, if relevant, needs to be taken into account when interpreting these changes.

This study has several limitations. First, its *in-vitro* design limits generalizability to the *in-vivo* setting. Even though trypsin-2 has been detected in the synovial fluid of rheumatoid arthritis-affected human joints^30^, unphysiological trypsin concentrations as well as culture and loading conditions limit the *in-vivo* correspondence of trypsin-induced proteoglycan depletion as studied in vitro. Moreover, cartilage degeneration as the hallmark change of OA is characterized by numerous structural and compositional changes beyond proteoglycan depletion, i.e. disruption and disorganisation of the collagen network amongst others^6^, which are not emulated in our trypsin-induced experimental setup of isolated proteoglycan depletion. This again limits the generalizability to the in vivo setting. Beyond, proteoglycan depletion may not have been consistent throughout the samples. Following preparation, the bare sample shoulders and periphery might have been more susceptible to trypsin, which might have affected the PPA more than the SPA. Second, the study’s reference framework only included histology, which may be too coarse to detect finer compositional tissue alterations^23^ and could be supplemented by more advanced techniques such as micro-spectroscopy, biochemical assaying techniques, polarized light microscopy, dGEMRIC or solid-state NMR in the future. In particular, solid-state NMR allows the quantitative evaluation of cartilage and its organic constituents at the molecular level with potential prospects as an additional ultrastructural reference^11^. Alongside larger sample sizes, more refined references are a prerequisite for more in-depth functional analysis of proteoglycan depletion, for example as a function of gender or age. In addition, very-high-resolution scanning probe methodologies, i.e. atomic force microscopy, scanning electron microscopy or transmission electron microscopy, could provide further insights into cartilage structure, composition, and biomechanical properties for reference purposes to complement quantitative MRI measurements^58^. Third, despite histological quality checks, the tissue source of total joint replacements is a potential confounder. Beyond the extent appreciable histologically, samples may have been pre-degenerated and pre-inflamed, thereby potentially contributing to sample heterogeneity as signified by considerable measures of spread such as standard deviations. Nonetheless, given the stark differences between animal and human cartilage^32,59^, we intended to obtain clinically meaningful results and deliberately included human articular cartilage to study its functionality in reference to the sample’s histological tissue status, which partially remedies this situation. Our experimental imaging setup, i.e. the clinical 3.0 T MRI scanner and the ready-to-use mapping sequences with clinically applicable parameter settings are also reflective of this intention. Nonetheless, truly healthy cartilage from amputations, organ donor networks or body donors ought to be included in future studies. Fourth, histological mismatching of the cartilage sample and adjacent tissue may have decreased validity and representatively of histological results, in particular with regards to the variable degeneration in joints, compartments, and tissue regions. Fifth, trypsin exposure was only terminated by continuous washing with PBS, while no dedicated trypsin inhibitors were used. Consequently, traces of trypsin may have remained in the samples, bringing about stronger-than-intended proteoglycan-depletive effects. Yet, this source of variability was systematic after LT and HT exposure, thereby not affecting this study’s main outcomes. Sixth, pre-exposure response-to-loading patterns were quite heterogeneous, in particular for T1ρ and T2, which has been observed before^18^. Even though principally attributable to the small sample size, it is important to realize that the tissue loading response is not just related to cartilage sample integrity and its primary or secondary degradation, but also to other person-level factors such as age, gender, obesity, and genetics, as well as joint-level factors such as axis alignment, muscle strength and other factors that are reflective of abnormal loading^60^. These variables are beyond our experimental control and may affect the tissue’s loading responses in health and disease to a much larger extent than previously thought. As detailed above, larger sample sizes with alternative tissue sources and stricter inclusion criteria may help clarify the contributions of these factors to cartilage functionality. Seventh, for the sake of comparability with literature data, we only performed conventional mono-exponential fitting. Alternative fitting techniques such as bi-exponential relaxation of T2^61^ help to quantify different water compartments in cartilage and may thus increase the specificity for altered hydration and functionality.

In conclusion, this study found that trypsin-induced proteoglycan depletion affects cartilage functionality as assessed by serial qMRI mapping and standardized pressure-controlled indentation loading. Dose-dependently, proteoglycan-depleted cartilage areas undergo substantially larger changes in T2 (and, to a lesser extent, T1) as an indication of the altered micromechanical environment secondary to deficient pre-stress of the collagen network. Simulating one of the earliest changes in cartilage degeneration, the close association between compositional deficit and altered functionality was not discernible from quantitative MR images in the unloaded configuration, thereby corroborating the diagnostic potential of imaging-based cartilage functionality assessment. Biomechanical imaging may provide a non-invasive means to study matrix changes in functional contexts and beyond mere composition and may perspectively advance joint and tissue diagnostics in patients.

## Materials and methods

### Study design

The present study was carried out as an intra-individual *ex-vivo* imaging study using human cartilage samples from the lateral femoral condyle that were harvested from total knee arthroplasties performed at the University Hospital Aachen. Prior to the study, local Institutional Review Board approval (Ethical Committee, RWTH Aachen, Germany, AZ-EK157/13) and individual written informed consent had been obtained. All methods were carried out in accordance with relevant guidelines and regulations.

### Preparation of cartilage samples

Following resection during surgery, the surgical material was transferred to the laboratory in sterile cell culture medium, i.e. Dulbecco’s modified Eagle’s medium (Thermo Fisher Scientific, Waltham, Massachusetts, US) that contained 10% fetal calf serum (PAN-Biotech, Aidenbach, Germany), 100 μg/mL Gentamycin (GIBCO-BRL, Paisley, UK), 100 U/mL penicillin and streptomycin (Sigma-Aldrich, St. Louis, Missouri, US), and 1.25 μg/mL amphotericin B (GIBCO-BRL). Surgical material from a total of 29 patients (mean age: 67.4 ± 8.8 years; range, 53–86 years; 13 males, 16 females) was included. Patients were only included if they had primary OA of the knee joint that involved at least the medial compartment, but excluded if they had other bone and joint conditions such as rheumatoid arthritis, secondary OA, and historical findings suggestive of previous trauma or surgery to the index knee. To avoid sample pooling, only one sample was obtained from each patient.

**Figure 4:**
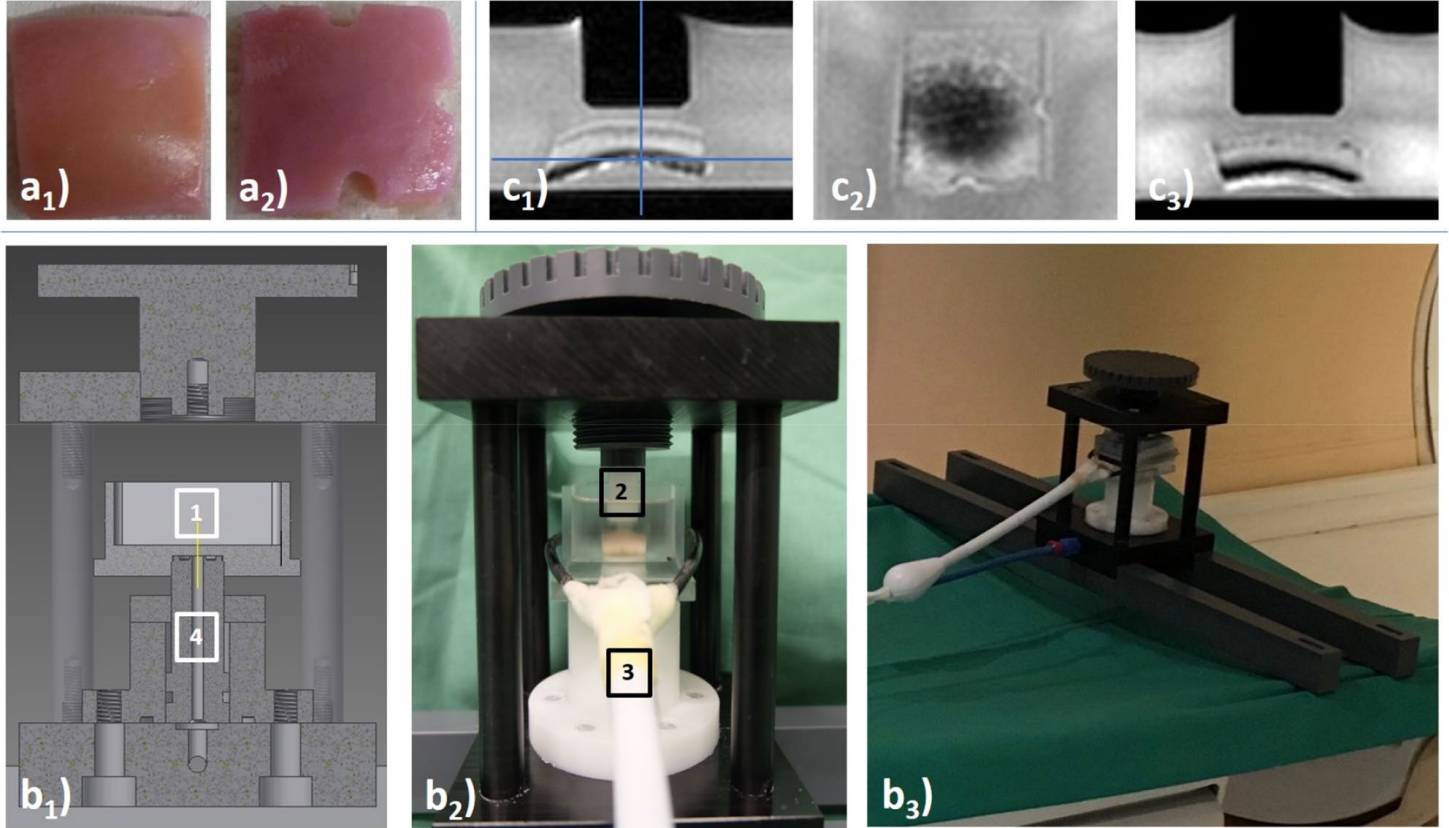
Details of cartilage preparation steps and experimental setup. (**a**) Top view of a representative osteochondral sample of standard square shape and size of 1.2 × 1.2 cm before (**a**_**1**_) and after (**a**_**2**_) creation of three notches to define the mid-sagittal imaging plane (extending from the 12 to the 6 o ‘clock position) and the sample centre (as the intersection of the mid-sagittal imaging plane and its orthogonal along the 3 o ‘clock position). (**b**) Cross-sectional construction sketch (**b**_**1**_) and photographs (lateral [**b**_**2**_] and angulated [**b**_**3**_] views) of the pressure-controlled indentation loading device outside (**b**_**2**_) and inside the MRI scanner (**b**_**3**_). Centrally positioned within the sample box (1), the cartilage sample was upwardly displaced against the indentor piston (2) that was adjustable in height. MR imaging was performed using a dedicated receiver coil that circumferentially comprised the sample box (3). Even though laid out to generate substantially greater forces, the pneumatic mechanism (4) was used to apply forces of 15.1 N and 28.6 N in the context of this study. For more details on the device, the interested reader is referred to Truhn et al., JMBBM, 2019^64^. (**c**) Sagittal (**c**_**1**_), axial (**c**_**2**_), and coronal (**c**_**3**_) Proton-Density-weighted images of a representative cartilage sample. Blue lines (in **c**_**1**_) indicate the respective heights of the axial (**c**_**2**_) and coronal (**c**_**3**_) images.

The surgical material was kept refrigerated at 4 °C for a maximum of 24 h before samples were subject to the following standardized preparation steps. First, the material was evaluated macroscopically according to the Outerbridge classification^62^, the central lateral femoral condyle was identified, and only intact joint regions without any gross macroscopic damage, i.e. Outerbridge grade 0, were prepared. Second, the material was cut to osteochondral samples of standard square shape and size of 1.2 × 1.2 cm (width × length) using dedicated rongeurs and scalpels. Third, we prepared the samples to be as even and plane as possible by preserving the subchondral lamella and by removing the cancellous bone underneath. By doing so, we intended to prevent loading-induced compaction and heterogeneity in load distribution despite the considerable curvature of the femoral condyles. Fourth, for reference purposes, two notches at opposing sample sides were created via rongeur to define the mid-sagittal plane, while an additional third notch was used to define the sample centre as the intersection of the mid-sagittal plane and its perpendicular along the third notch (Fig. 4a). In line with the orientation of the native human knee joint, the sample’s midsagittal plane was aligned along the main magnetic field B_0_ within the sample box that was subsequently filled with PBS (phosphate-buffered solution, Sigma-Aldrich). Fifth, directly adjacent cartilage tissue with similarly grossly intact macroscopic appearance was prepared to determine the histological status of the sampled cartilage region before any intervention (see Histological analysis below).

Prior to this study, sample size had been estimated by use of a dedicated online tool (https://www.statstodo.com). Based on earlier literature and our own experimental data^17,18,43,63^, we estimated a minimum sample size of 28 with a statistical power of 0.9, a probability of type-I error (α) of 0.05, a maximum inter-group difference of 0.6, an intra-group standard deviation of 0.5, and Cohen’s effect size model f2. Accordingly, we included a total of 29 macroscopically intact cartilage samples.

### MRI-compatible device for standardized indentation loading

The device has been described and validated earlier^64^ and is presented synoptically in Fig. 4b. In brief, the setup consists of a loading unit inside the MRI scanner’s bore and an outside control unit. Both units are connected via standard pressure lines (Festo, Esslingen, Germany). Customized software routines implemented in LabVIEW software (v2017, National Instruments Corporation, Austin, US) are used to electronically actuate a high-precision valve (± 0.01 bar, Type: VPPM-6L-L-1-G18–0L6H-V1P-S1C1, Festo) via a digital-to-analogue converter (Multifunction I/O USB-6001, National Instruments Corporation). Maximum pressure (up to 4.69 bar) is provided by the hospital’s in-house pressure system and thus downregulated to pressures of 0.75 bar and 1.5 bar that are equivalent to forces of 15.1 N and 28.6 N, respectively^64^. These forces are approximately equivalent to the forces experienced in vivo during two-legged stance^65^. Once the desired pressure level is set, the pneumatic mechanism contained within the device’s framework displaces the firmly attached sample box upwards against the fixed and non-porous indentor piston (diameter: 10 mm, bevel: 1 mm) attached to the device’s upper frame. The centrally positioned cartilage sample thus experiences standardized indentation loading by control of pressure.

### MRI measurements prior to exposure

Following preparation, pre-exposure MRI measurements were performed on a clinical 3.0 T MRI scanner (Achieva, Philips, Best, The Netherlands). A modified single channel receive-only prostate coil (BPX-30 disposable endorectal coil, Medrad/Bayer, Germany) without the inflatable balloon tip that circumferentially comprised the transparent sample box at the height of the cartilage layer was used for imaging. The scanner’s in-built body coil was used for the application of the radiofrequency pulses. Cartilage samples were centred underneath the indentor piston and attention was paid to align the piston’s undersurface with the sample’s surface.

Imaging of the cartilage samples was performed individually and serially, i.e. in the unloaded configuration (δ_0_) followed by two consecutive loading positions, i.e. at 0.75 bar (δ_1_) and at 1.5 bar (δ_2_). After scout views, Proton Density-weighted (PDw) sequences in all three planes (Fig. 4c) and T2*, T2, T1ρ, and T1 mapping sequences of the mid-sagittal plane were obtained in this order. Table 3 gives the detailed imaging protocol. Of note, the imaging protocol was completed for each individual sample and loading position. After setting pressure levels to 0.75 bar or 1.5 bar, we observed an equilibration period of 5 min prior to initiating the measurements. PDw sequences were assessed to confirm the absence of sample displacement and, thus, adequate loading prior to the acquisition of the mapping sequences. Moreover, PDw sequences were used to guide the mid-sagittal imaging plane along the mid-sagittal plane as defined by the notches. Per sample and loading position, scanner time was about 40 min, equaling 140 min per sample and measurement series (δ_0_–δ_2_). Imaging was performed at room temperature which was monitored during one measurement series (20.1 ± 0.6 °C).

**Table 3:** Acquisition parameters of MR sequences. n/a—not applicable, ax—axial, sag—sagittal, cor—coronal, mid-sag—mid-sagittal. *Indicates the total duration of all three sequences.

	PDW	T1	T1ρ	T2	T2*
Sequence type	Turbo-spin echo (2D)	Inversion-recovery (2D)	Spin-lock multi-gradient echo (3D)	Multi-spin echo (2D)	multi-gradient echo (2D)
Orientation	ax, sag, cor	mid-sag	sag	mid-sag	mid-sag
Repetition time (ms)	1,500	3,000	30	1,500	700
Echo time (ms)	11.2	10.1	3.8	n × 8.4 (n = 1–12)	3.3 + n × 5.2 (n = 0–14)
Turbo spin-echo factor	6	5	44	12	15
Field of view (mm)	62 × 62	62 × 62	52 × 52	52 × 52	52 × 52
Acquisition matrix	144 × 142	224 × 220	176 × 176	176 × 176	176 × 176
Reconstruction matrix	256 × 256	224 × 224	224 × 224	224 × 224	224 × 224
Flip angle (°)	90	90	11	90	90
Number of signal averages	2	1	4	2	3
Slices	10	1	7	1	1
Slice Thickness / Gap (mm)	1.0 / 0.5	2.0 / n/a	3.2	2.0 / n/a	2.0 / n/a
Inversion times (ms)	n/a	150, 300, 500, 800, 1,000, 1,500	n/a	n/a	n/a
Spin-lock durations (ms)	n/a	n/a	0, 10, 20, 30, 40	n/a	n/a
Duration (min:s)	7:03 (*)	7:12	14:30	4:29	03:08

### Pathomimetic trypsin model

Following completion of the pre-exposure MRI measurement series, the cartilage samples were randomly allocated to one of three study arms, #1) trypsin at high concentration (1.0 mg/mL)—high trypsin (HT) exposure (n = 10), #2) trypsin at low concentration (0.1 mg/mL)—low trypsin (LT) exposure (n = 10), and #3) controls without any trypsin exposure (n = 9).

Practically, samples were individually suspended in 50 mL volume tubes (Falco Tube, Sarstedt, Nümbrecht, Germany) that contained.

- *for study arm #1* 1.6 mL trypsin-Ethylenediaminetetraacetic acid (EDTA) solution (T4174, Sigma-Aldrich) and 6.4 mL sterile PBS to obtain the high trypsin concentration (1.0 mg/mL trypsin);
- *for study arm #2* 0.16 mL trypsin–EDTA solution and 7.84 mL sterile PBS to obtain the low trypsin concentration (0.1 mg/mL trypsin);
- *for study arm #3* 8 ml medium plus additives as detailed above to obtain controls.

Trypsin was obtained as a 10 × solution containing 5.0 g/L porcine trypsin (from porcine pancreas cells) and 2.0 g/L EDTA. Trypsin is commonly used in pathomimetic models of articular cartilage to induce proteoglycan depletion and the chosen concentrations of 0.1 mg/mL and 1.0 mg/mL are widely consented in the literature^23–25^. Even though these concentrations are unphysiological, they are commonly used to realize rapid proteoglycan degradation. By means of a tube rotator (VWR Tube Rotator, VWR, Amsterdam, The Netherlands), tubes were continuously rotated for 2 h while being incubated in a standard laboratory incubation unit at 37 °C, 5% CO_2_, and 21% O_2_. After incubation, proteolysis was halted by discarding the trypsin–EDTA solutions and by continuous washing with sterile PBS for 1 h, after which samples were replaced in medium + additives as above.

### MRI measurements after exposure

Within 24 h after the pre-exposure MRI measurements, cartilage samples were imaged again in strict analogy to the first measurement conditions. While trying to align pre- and post-exposure framework conditions, we were particularly careful to realize identical sample orientation and position.

### Image analysis and post processing

#### Cartilage sample height

Sample height was determined on the mid-sagittal PDw images for each cartilage sample and loading position individually. The first author (TH with 2 years of experience in musculoskeletal radiology) measured cartilage sample height at the sample centre and at an equidistance of 2 mm to both sides by use of the digital caliper tool of the in-house PACS (Picture Archiving and Communication System, Philips). With a step size of a single pixel, the caliper tool has a resolution of 0.24 × 0.24 mm.

### Quantitative T1, T1ρ, T2 and T2* maps

**Figure 5:**
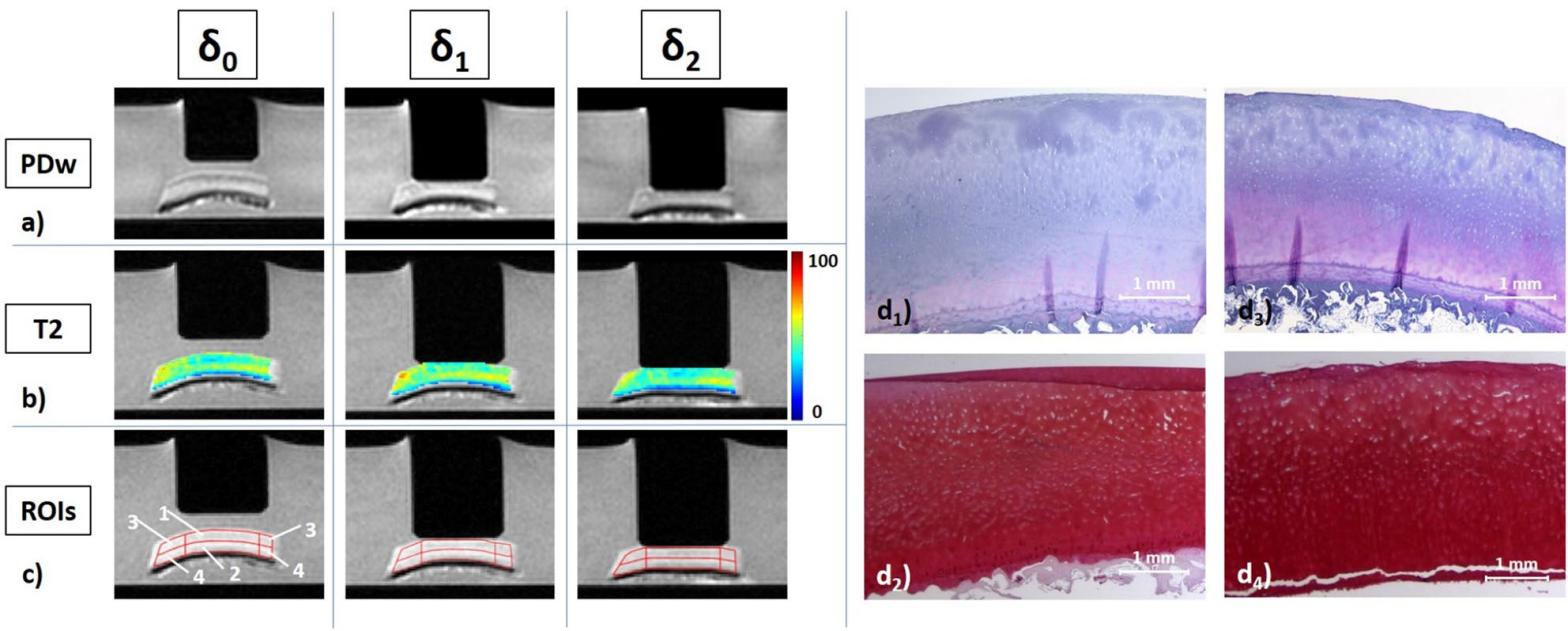
Details of image analysis, post processing, and histological referencing. (**a**) Mid-sagittal Proton Density-weighted images of a representative cartilage sample subject to increasing loading intensity, i.e. at the unloaded configuration (δ_0_) and under loading to 15.1 N (δ_1_), and 28.6 N (δ_2_). (**b**) Corresponding T2 maps overlaid onto the respective morphological images (TE 41.9 ms). Unit of scale on the right is [ms]. (**c**) Regional and zonal ROIs were defined as the upper and lower sample halves as well as the sub-pistonal (SPA) and peri-pistonal area (PPA). Consequently, seven ROIs were evaluated: (1) SPA_upper_, (2) SPA_lower_, (3) PPA_upper_, (4) PPA_lower_, (5) SPA full thickness (1 + 2), (6) PPA full thickness (3 + 4), (7) entire cartilage sample (ECS = 1 + 2 + 3 + 4). (**d**) Corresponding histological sections, stained with hematoxylin/eosin (**d**_**1**_,**d**_**3**_) and Safranin O (**d**_**2**_,**d**_**4**_), of the cartilage sample following incubation without enzyme exposure and pre- and post-exposure measurements (**d**_**1**_,**d**_**2**_) and of the adjacent cartilage tissue at the time of sample preparation (**d**_**3**_,**d**_**4**_). Same cartilage sample as in Fig. 4 (control).

First, image raw data were imported and respective time constants for each pixel of the mid-sagittal image were determined based on customized mono-exponential fitting routines implemented in Matlab (MatlabR2019a, Natick, USA) to generate spatially resolved quantitative T1, T1ρ, T2 and T2* maps as before^17,20^. Fit quality was checked using R^2^ statistics adjusted to the degrees of freedom and only pixel with R^2^ values > 0.95 were included. For T2 and T2* fitting, we only included echo times < 60 ms to decrease the effects of noise. Second, cartilage tissue was segmented by manual definition of the sample outlines. While considering the corresponding mid-sagittal PDw image, T2-weighted morphological image (of echo time 41.9 ms), and histological sections as reference, the first author (TH) performed the segmentations of each cartilage sample at each loading position. Segmentation was performed conservatively to decrease partial volume effects by only including pixels that safely lay inside the cartilage tissue and by excluding boundary pixels at the topmost (towards piston or medium) and lowermost layer (towards subchondral lamella). Third, the sample- and loading position-specific outlines were validated against the corresponding T1, T1ρ, T2 and T2* maps and quality checked for morphological correspondence by visual inspection (DT and SN, each with 8 years of experience in musculoskeletal radiology) (Fig. 5a,b). Fourth, using a customized algorithm implemented in Matlab, distinct regions-of-interest (ROIs) were automatically defined on the mid-sagittal images. Zonal ROIs were obtained by automatically partitioning the sample outlines into two equal layers based on pixel-wise measurements of cartilage sample height to halve the entire cartilage sample (ECS) in two halves of equal height, i.e. the superficial (‘upper’) and deep (‘lower’) halves. Regional ROIs were defined in relation to the indentor piston as the (i) sub-pistonal area (SPA, defined as interface between the piston’s undersurface and the sample’s surface of 8 mm width) and the ii) peri-pistonal area (PPA, defined as the tissue region that was located bilaterally adjacent to the SPA). Both bilateral PPA regions were merged, resulting in a total of seven distinct ROIs for downstream analyses: (1) entire cartilage sample (ECS), (2) SPA full thickness (SPA), (3) PPA full thickness (PPA), (4) SPA superficial half (SPA_upper_), (5) SPA deep half (SPA_lower_), (6) PPA superficial (PPA_upper_), and (7) PPA deep (PPA_lower_) (Fig. 5c).

### Histological analysis

In line with standard procedures, the cartilage samples and the adjacent cartilage tissue were subject to histological processing and analysis. After simultaneous decalcification and fixation in Ossa fixona (Waldeck, Muenster, Germany), cartilage samples were sectioned along the mid-sagittal plane, while the adjacent tissue was sectioned parallel to it. Following sectioning, the material was embedded in paraffin, cut to 5-µm sections, stained with hematoxylin/eosin and Safranin O, and imaged using a standard light microscope (Leica DM/LM-P, Wetzlar, Germany) (Fig. 5d).

Histological sections were analysed by three blinded investigators experienced in musculoskeletal histopathology (TH [MD, 2 years of experience], MP [MD, 3 years of experience], SN [MD, fellowship trained, 11 years of experience]) who performed the histological evaluation individually. Adjacent cartilage tissue was assessed in terms of histological degeneration to quality check the included material. To this end, semi-quantitative grading of degeneration was performed according to the Mankin classification^66^ by assessment of tissue structure (score 0–6), cellularity (score 0–3), proteoglycan staining intensity (score 0–4), and tidemark integrity (score 0–1). Based on the resultant Mankin sum score (ranging from 0–14, i.e. no degeneration to most severe degeneration), the adjacent cartilage tissue was grouped into Mankin grades, i.e. MG 0 (Mankin sum scores 0–4), MG I (scores 5–8), MG II (scores 9–10), and MG III (scores 11–14)^67^. Only if initial scores were different between the investigators were individual sections discussed until consensus was established.

Cartilage samples following incubation with or without additional trypsin exposure were evaluated qualitatively with a focus on structural and compositional changes as compared to the adjacent tissue.

### Statistical analysis

The first and last authors (TH, SN) carried out the statistical analyses using GraphPad Prism (Version 7.0; GraphPad, San Diego, CA, USA). As detailed above, δ_0_ refers to the unloaded reference configuration, while δ_1_ and δ_2_ refer to moderate and strong loading configurations, while the relative changes at δ_1_ or δ_2_ versus δ_0_ are referred to as Δ_1_ or Δ_2_. For a given qMRI parameter Tx, Δ_x_ was calculated as Δ_x_ = ((Tx_δx_/Tx_δ0_) − 1) * 100 [%]. Analogously, relative changes between the unloaded pre- and post-exposure configurations δ_0post_ vs. δ_0pre_ were defined as Δ_0_ and calculated as Δ_0_ = ((Tx_δ0post_/Tx_δ0pre_) − 1) * 100 [%]. Throughout, ROI-specific analyses were performed as a function of loading intensity and study arm. To evaluate loading-induced changes in the samples prior to as compared to after incubation with or without additional trypsin exposure, pair-wise comparisons of pre- and post-exposure measures, i.e. Δ_1pre_ vs. Δ_1post_, and Δ_2pre_ vs. Δ_2post_, were performed by paired Student's *t* tests. As absolute T1, T1ρ, T2, and T2* values were not assumed to be normally distributed, they were analysed using Friedman's test followed by Dunn's post-hoc test. To assess changes in the unloaded reference configuration because of incubation with or without additional trypsin exposure, δ_0pre_ and δ_0post_ were comparatively evaluated using the Wilcoxon matched pairs test. To assess loading-induced changes in the samples’ cross-sectional areas, sample heights and pixel numbers were analysed by repeated measures ANOVA. Supplementary Figure S2 graphically presents the most important comparisons.

Data are presented as median and interquartile range (for non-normally distributed data) or mean ± standard deviation (for normally distributed data). To account for the multiple comparisons performed in this exploratory study and control the family-wise error rate, Bonferroni-Holm correction was used. À priori, the level of significance was set to *p* ≤ 0.01 to contain the number of statistically significant, yet scientifically (likely) insignificant findings.

## Supplementary information

Supplementary Information.

Supplementary Legends.

## Data Availability

The datasets generated and analyzed in this study are available from the corresponding author on reasonable request.

## Abbreviations

ECM: Extracellular matrix
ECS: Entire cartilage sample
EDTA: Ethylenediaminetetraacetic acid
HT: High concentration of trypsin
LT: Low concentration of trypsin
MG: Mankin Grade
OA: Osteoarthritis
PBS: Phosphate-buffered solution
PDw: Proton Density-weighted
PPA: Peri-pistonal area
(q)MRI: (Quantitative) Magnetic Resonance Imaging
ROI: Region-of-interest
SPA: Sub-pistonal area
Δ: Relative change
δ_0_: Reference configuration
δ_x_: Measurement configuration x
