# Supplementary material for: A serial multiparametric quantitative magnetic resonance imaging study to assess proteoglycan depletion of human articular cartilage and its effects on functionality
Source: Sci Rep. 2020 Sep 15;10:15106. doi: 10.1038/s41598-020-72208-y (PMC7492285; doi:10.1038/s41598-020-72208-y)

Title: A Serial Multiparametric Quantitative Magnetic Resonance Imaging Study to Assess Proteoglycan Depletion of Human Articular Cartilage and Its Effects on Functionality

Authors: Tobias Hafner[1§], Justus Schock[2,3§], Manuel Post[1], Daniel Benjamin Abrar[2], Philipp Sewerin[4], Kevin Linka[5], Matthias Knobe[6], Christiane Kuhl[1], Daniel Truhn[1], Sven Nebelung[2*]

Affiliations: 1 Aachen University Hospital, Department of Diagnostic and Interventional Radiology, Aachen, Germany
2 University Dusseldorf, Medical Faculty, Department of Diagnostic and Interventional Radiology, D-40225 Dusseldorf, Germany
3 Institute of Computer Vision and Imaging, RWTH University Aachen, Aachen, Germany
4 University Dusseldorf, Medical Faculty, Department and Hiller-Research-Unit for Rheumatology, Dusseldorf, Germany
5 Hamburg University of Technology, Department of Continuum and Materials Mechanics, Hamburg, Germany
6 Clinic for Orthopaedic and Trauma Surgery, Cantonal Hospital Luzern, Luzern, Switzerland.

Journal: Scientific Reports

Supplementary Table S1 (online only)

	**pre exposure**				**post exposure**			
	$\boldsymbol{\delta_{0pre}}$	$\boldsymbol{\delta_{1pre}}$	$\boldsymbol{\delta_{2pre}}$	**p-value**	$\boldsymbol{\delta_{0post}}$	$\boldsymbol{\delta_{1post}}$	$\boldsymbol{\delta_{2post}}$	**p-value**
**low trypsin**	2.59 ± 0.32	1.72 ± 0.30 (-34 ± 6 %)	1.50 ± 0.36 (-43 ± 9 %)	**< 0.001**	2.55 ± 0.31 (†)	1.78 ± 0.36 (-30 ± 9 %)	1.52 ± 0.38 (-41 ± 10 %)	**< 0.001**
**high trypsin**	2.40 ± 0.41	1.71 ± 0.30 (-28 ± 8 %)	1.64 ± 0.22 (-31 ± 9 %)	**< 0.001**	2.43 ± 0.41 (‡)	1.71 ± 0.30 (-29 ± 8 %)	1.53 ± 0.19 (-36 ± 8 %)	**< 0.001**
**controls**	2.77 ± 0.19	2.07 ± 0.26 (-26 ± 6 %)	1.86 ± 0.37 (-34 ± 9 %)	**< 0.001**	2.66 ± 0.22 (§)	2.00 ± 0.34 (-25 ± 10 %)	1.82 ± 0.41 (-32 ± 11 %)	**< 0.001**

Supplementary Table S2 (online only)

		pre exposure			post exposure			
		δ_{0pre}	δ_{1pre}	δ_{2pre}	δ_{0post}	δ_{1post}	δ_{2post}	**p-value**
**low trypsin**	**ECS**	344.4 ± 72.3	339.2 ± 70.7	307.7 ± 68.3	348.3 ± 56.5	323.2 ± 67.1	303.3 ± 62.4	**< 0.001**
	**SPA**	215.8 ± 47.9	208.9 ± 47.4	186.0 ± 45.4	218.2 ± 39.8	200.0 ± 44.4	181.4 ± 40.6	**< 0.001**
	**SPA upper**	107.6 ± 24.8	104.3 ± 25.3	93.6 ± 26.5	109.5 ± 19.5	100.2 ± 22.2	90.8 ± 18.1	**< 0.001**
	**SPA lower**	108.2 ± 23.5	104.6 ± 22.7	92.4 ± 20.2	108.7 ± 20.7	99.8 ± 22.9	90.6 ± 23.7	**< 0.001**
	**PPA**	124.7 ± 30.2	125.1 ± 29.1	118.8 ± 28.1	123.0 ± 27.2	117.9 ± 31.2	118.8 ± 29.9	0.088
	**PPA upper**	67.8 ± 16.1	66.5 ± 13.4	63.5 ± 13.8	66.0 ± 14.2	63.2 ± 16.3	61.1 ± 16.6	**0.004**
	**PPA lower**	56.9 ± 14.2	58.6 ± 15.8	55.3 ± 14.9	57.0 ± 13.3	54.7 ± 15.1	57.7 ± 14.0	0.392
**high trypsin**	**ECS**	281.3 ± 67.5	263.5 ± 51.5	253.0 ± 62.2	289.4 ± 68.9	258.5 ± 60.9	235.8 ± 61.9	**< 0.001**
	**SPA**	185.6 ± 44.7	173.1 ± 34.5	164.1 ± 39.9	193.7 ± 41.9	170.2 ± 39.2	148.8 ± 38.5	**< 0.001**
	**SPA upper**	94.5 ± 22.0	89.2 ± 17.2	82.6 ± 19.3	97.1 ± 21.0	82.7 ± 21.2	75.2 ± 20.0	**< 0.001**
	**SPA lower**	91.1 ± 23.2	83.9 ± 18.3	81.5 ± 22.6	96.6 ± 21.3	87.5 ± 18.3	73.6 ± 20.9	**< 0.001**
	**PPA**	92.6 ± 28.5	89.9 ± 25.6	85.3 ± 25.8	93.8 ± 34.8	85.2 ± 26.1	84.6 ± 28.4	0.017
	**PPA upper**	49.7 ± 15.2	48.8 ± 13.3	45.6 ± 14.3	51.7 ± 19.9	46.3 ± 14.8	44.5 ± 14.6	**0.009**
	**PPA lower**	42.9 ± 13.7	41.1 ± 12.9	39.7 ± 11.8	42.1 ± 15.3	38.9 ± 11.5	40.1 ± 14.0	0.151
**controls**	**ECS**	307.3 ± 52.3	293.5 ± 50.6	273.7 ± 45.5	306.0 ± 51.3	298.9 ± 46.3	273.8 ± 50.5	**< 0.001**
	**SPA**	216.1 ± 32.0	202.7 ± 27.6	188.6 ± 25.8	216.0 ± 31.1	206.3 ± 27.4	189.1 ± 35.1	**< 0.001**
	**SPA upper**	106.3 ± 17.5	103.6 ± 14.5	96.4 ± 12.1	107.5 ± 14.4	104.0 ± 15.4	93.3 ± 17.0	**< 0.001**
	**SPA lower**	109.8 ± 15.0	99.1 ± 14.8	92.2 ± 14.7	108.5 ± 17.0	102.3 ± 15.6	95.8 ± 18.5	**< 0.001**
	**PPA**	88.8 ± 28.9	88.4 ± 32.2	83.2 ± 27.5	89.6 ± 29.2	91.5 ± 25.9	84.3 ± 23.9	0.043
	**PPA upper**	49.7 ± 15.2	48.8 ± 13.3	45.6 ± 14.3	51.7 ± 19.9	46.3 ± 14.8	44.5 ± 14.6	0.019
	**PPA lower**	41.0 ± 15.2	40.8 ± 15.6	41.1 ± 15.9	42.4 ± 15.6	43.7 ± 15.0	40.9 ± 13.6	0.218

Supplementary Table S3 (online only)

			pre exposure				post exposure			
			δ_{0pre}	δ_{1pre}	δ_{2pre}	p-value	δ_{0post}	δ_{1post}	δ_{2post}	p-value
**T1**	**low trypsin**	**entire**	658.0 (646.0-692.7)	640.3 (632.5-654.2)	**618.8 (604.6-628.5)**	**<0.001**	672.9 (663.4-686.2)	651.0 (635.9-666.3)	**628.6 (601.2-650.8)**	**<0.001**
		**SPA**	658.0 (647.8-691.7)	624.3 (613.4-644.8)	**598.5 (585.6-613.7)**	**<0.001**	664.6 (657.5-685.6)	634.6 (621.7-656.0)	**603.6 (579.8-613.2)**	**<0.001**
		**SPA upper**	739.1 (724.9-774.6)	682.1 (659.5-704.3)	**643.5 (589.6-652.0)**	**<0.001**	751.3 (733.0-781.0)	691.0 (679.9-698.6)	**631.5 (609.1-652.0)**	**<0.001**
		**SPA lower**	582.4 (572.0-624.8)	572.6 (553.7-603.0)	574.1 (569.2-582.2)	0.046	585.1 (574.8-606.2)	582.4 (563.2-599.2)	575.4 (567.7-578.0)	0.066
		**PPA**	660.2 (649.9-692.4)	665.6 (649.2-673.6)	**647.3 (641.7-653.6)**	**0.006**	681.2 (671.7-689.7)	681.0 (655.3-687.4)	662.5 (635.6-684.2)	0.135
		**PPA upper**	727.4 (713.1-758.2)	734.2 (705.9-747.4)	**711.3 (679.8-724.9)**	**<0.001**	749.7 (737.2-773.7)	749.0 (731.7-757.6)	726.9 (681.0-752.2)	0.026
		**PPA lower**	583.4 (569.4-635.5)	593.1 (587.1-601.5)	578.5 (575.2-595.7)	0.066	600.0 (584.5-626.3)	599.6 (583.6-614.3)	592.2 (570.5-620.3)	0.601
	**high trypsin**	**entire**	649.6 (627.5-731.9)	627.9 (612.1-691.9)	**599.2 (584.0-643.6)**	**<0.001**	662.4 (650.0-746.9)	627.4 (619.4-695.6)	**600.0 (581.5-651.6)**	**<0.001**
		**SPA**	651.1 (626.5-729.6)	624.9 (599.8-678.9)	**587.0 (571.1-618.0)**	**<0.001**	658.1 (649.0-746.9)	616.3 (584.9-670.2)	**583.8 (552.8-616.0)**	**<0.001**
		**SPA upper**	724.0 (706.9-819.2)	688.0 (661.9-730.9)	**631.1 (617.9-649.2)**	**<0.001**	783.7 (717.0-848.4)	676.2 (649.1-707.4)	**608.2 (599.6-633.7)**	**<0.001**
		**SPA lower**	586.9 (554.7-639.2)	564.4 (531.3-627.8)	**542.3 (517.1-586.9)**	**0.003**	589.1 (537.9-645.3)	587.8 (545.0-626.1)	**565.3 (516.3-596.6)**	**0.006**
		**PPA**	651.9 (635.5-750.5)	648.0 (629.3-715.2)	**630.9 (608.7-677.7)**	**<0.001**	676.6 (649.6-754.7)	674.5 (645.2-733.4)	**634.2 (626.3-698.4)**	**<0.001**
		**PPA upper**	743.1 (714.3-811.9)	744.4 (690.4-775.6)	**714.8 (669.4-729.0)**	**<0.001**	794.1 (733.2-845.9)	765.4 (735.3-793.5)	**736.4 (700.4-756.4)**	**<0.001**
		**PPA lower**	577.9 (543.8-641.0)	569.3 (551.8-619.6)	**562.3 (539.9-602.5)**	**<0.001**	559.2 (536.0-632.5)	597.7 (532.8-639.2)	572.0 (531.2-634.0)	0.135
	**controls**	**entire**	679.3 (621.5-687.4)	653.5 (592.1-664.3)	**617.3 (567.1-632.0)**	**<0.001**	672.5 (629.5-677.3)	634.3 (595.7-647.7)	**596.5 (572.2-619.9)**	**<0.001**
		**SPA**	670.4 (614.0-682.4)	645.7 (583.0-655.4)	**612.1 (548.6-624.8)**	**<0.001**	665.4 (630.4-668.4)	615.6 (577.7-636.7)	**588.8 (552.8-605.7)**	**<0.001**
		**SPA upper**	735.9 (702.5-769.5)	679.7 (660.0-717.7)	**626.5 (616.9-667.8)**	**<0.001**	724.0 (706.5-747.6)	650.7 (624.8-695.9)	**618.0 (598.3-638.7)**	**<0.001**
		**SPA lower**	573.6 (549.1-609.9)	569.9 (551.5-609.4)	552.0 (523.0-600.0)	0.057	575.9 (549.5-594.0)	552.2 (533.9-586.3)	550.2 (508.4-586.2)	0.107
		**PPA**	680.6 (642.6-695.5)	666.1 (617.8-671.2)	**628.3 (596.3-651.6)**	0.019	666.1 (631.1-694.5)	643.4 (627.2-686.1)	**618.3 (603.3-638.1)**	**0.003**
		**PPA upper**	748.7 (710.7-749.6)	705.6 (691.3-744.7)	**675.7 (650.6-706.2)**	**0.006**	726.9 (714.9-765.7)	709.3 (674.2-743.1)	**662.3 (645.2-691.7)**	**<0.001**
		**PPA lower**	581.0 (564.1-605.2)	576.3 (560.7-622.7)	572.7 (543.4-593.4)	0.814	587.0 (565.1-590.5)	572.0 (549.7-605.2)	567.6 (536.6-597.0)	0.685
**T1ρ**	**low trypsin**	**entire**	62.7 (58.4-66.2)	**72.0 (66.6-73.2)**	**72.2 (67.9-75.2)**	**<0.001**	60.7 (57.7-69.0)	**68.4 (65.5-76.6)**	**70.4 (66.5-73.6)**	**<0.001**
		**SPA**	65.2 (60.1-69.4)	**76.3 (70.1-77.8)**	**74.4 (72.6-78.4)**	**<0.001**	63.9 (60.5-70.0)	**72.7 (67.8-79.6)**	**74.3 (69.7-76.7)**	**<0.001**
		**SPA upper**	68.7 (64.8-72.9)	**83.7 (77.8-88.6)**	**80.8 (78.1-87.8)**	**<0.001**	70.9 (61.9-73.1)	**83.6 (74.6-88.1)**	**82.4 (75.3-84.4)**	**<0.001**
		**SPA lower**	59.1 (54.7-65.4)	65.8 (61.2-69.6)	**68.2 (64.0-69.6)**	**0.002**	59.5 (55.9-64.7)	64.1 (60.0-71.1)	64.6 (62.5-69.6)	0.222
		**PPA**	59.2 (54.3-62.5)	63.3 (62.4-67.4)	**65.2 (62.4-68.7)**	**<0.001**	57.1 (50.7-67.5)	**60.5 (59.7-70.4)**	**61.9 (61.0-68.3)**	**<0.001**
		**PPA upper**	60.0 (57.3-65.1)	**70.1 (66.2-71.3)**	**70.5 (67.5-74.0)**	**<0.001**	60.6 (51.8-68.0)	68.2 (62.7-76.6)	68.2 (64.8-73.7)	**0.007**
		**PPA lower**	54.5 (50.7-60.5)	58.3 (55.3-62.2)	**58.9 (56.8-64.4)**	**0.003**	55.0 (48.0-62.4)	56.0 (51.8-62.8)	57.1 (55.0-60.8)	0.046
	**high trypsin**	**entire**	62.7 (55.2-64.9)	**68.4 (64.9-71.3)**	**69.2 (65.8-72.3)**	**<0.001**	62.3 (56.7-70.2)	**67.0 (64.0-74.5)**	69.2 (64.3-76.0)	**0.006**
		**SPA**	64.4 (55.5-66.3)	**71.4 (68.0-73.0)**	**70.6 (68.6-75.0)**	**<0.001**	64.6 (60.7-70.4)	**70.5 (67.5-76.5)**	71.1 (68.5-76.8)	**0.003**
		**SPA upper**	69.0 (59.5-74.3)	**79.9 (70.2-83.1)**	78.6 (72.3-83.8)	**<0.001**	71.7 (64.4-81.5)	**78.5 (70.1-83.8)**	77.2 (73.0-84.9)	**0.003**
		**SPA lower**	58.0 (53.2-58.2)	64.2 (60.7-67.2)	**68.1 (61.5-69.0)**	**<0.001**	57.5 (54.5-59.6)	66.3 (61.3-68.1)	67.4 (63.6-68.4)	**0.007**
		**PPA**	57.4 (53.7-64.0)	61.4 (59.0-66.8)	63.0 (59.3-69.8)	0.030	56.6 (49.8-70.2)	62.0 (56.3-71.3)	62.8 (58.8-70.1)	0.135
		**PPA upper**	65.3 (56.3-67.5)	67.0 (60.8-74.1)	**68.8 (63.4-73.7)**	0.012	65.3 (52.3-72.9)	65.9 (59.4-78.9)	66.7 (60.8-75.1)	0.078
		**PPA lower**	53.9 (48.7-57.7)	56.5 (54.9-61.5)	60.8 (55.3-63.1)	0.222	52.0 (44.4-60.3)	56.4 (52.7-63.4)	**58.2 (54.8-66.5)**	**0.003**
	**controls**	**entire**	65.1 (61.3-72.6)	71.5 (69.9-79.1)	71.1 (70.3-78.4)	0.016	59.8 (52.5-66.8)	67.1 (63.2-71.1)	**68.6 (66.0-71.5)**	**<0.001**
		**SPA**	68.2 (62.7-73.2)	**74.9 (71.8-81.8)**	**74.8 (72.7-80.4)**	**<0.001**	59.9 (53.2-66.5)	70.1 (64.3-72.8)	**71.2 (67.6-73.5)**	**<0.001**
		**SPA upper**	72.2 (66.2-79.2)	**82.2 (79.2-90.7)**	**86.3 (77.1-88.6)**	**<0.001**	64.8 (57.0-71.1)	**77.5 (70.3-82.5)**	**76.7 (74.1-82.0)**	**<0.001**
		**SPA lower**	64.4 (57.7-70.2)	66.9 (64.4-70.4)	**67.8 (64.8-72.9)**	**0.006**	55.1 (49.5-59.0)	60.5 (56.0-63.5)	**62.1 (60.4-65.7)**	0.019
		**PPA**	62.3 (57.9-70.6)	65.0 (62.7-73.3)	65.0 (63.5-72.3)	0.031	59.0 (49.5-63.0)	61.9 (54.9-66.5)	**60.9 (60.1-65.7)**	**0.010**
		**PPA upper**	66.9 (61.6-73.7)	69.7 (66.1-79.8)	69.4 (65.7-77.8)	0.107	64.0 (50.2-67.4)	**69.4 (60.3-69.7)**	**66.0 (61.3-71.2)**	**<0.001**

		**PPA lower**	58.2 (57.5-65.8)	62.5 (58.7-64.5)	62.5 (61.3-64.4)	0.278	54.1 (46.3-58.4)	55.4 (51.2-58.8)	**58.4 (53.8-59.3)**	**0.006**
**T2**	**low trypsin**	**entire**	37.6 (35.3-42.3)	40.6 (38.4-44.7)	41.1 (38.8-42.8)	0.018	40.0 (37.9-45.3)	41.0 (37.6-46.2)	41.7 (38.0-44.4)	0.135
		**SPA**	36.8 (34.5-43.4)	40.0 (37.0-45.4)	39.8 (38.4-42.4)	0.030	40.9 (36.5-43.6)	41.1 (36.0-44.9)	41.9 (36.4-43.1)	0.135
		**SPA upper**	44.3 (43.1-49.1)	48.1 (46.4-51.9)	46.6 (44.4-50.3)	0.066	50.4 (43.1-53.8)	50.0 (41.8-54.6)	46.7 (42.3-50.2)	0.092
		**SPA lower**	30.5 (25.8-39.1)	31.1 (28.1-41.6)	31.6 (30.8-37.2)	0.316	32.6 (28.9-36.6)	33.5 (28.2-40.1)	34.9 (29.7-40.5)	0.316
		**PPA**	38.7 (36.5-41.9)	**41.7 (39.4-43.1)**	**42.8 (38.8-43.7)**	**0.001**	40.9 (36.2-47.6)	41.5 (38.4-49.0)	43.0 (40.4-47.4)	0.187
		**PPA upper**	42.6 (41.1-49.6)	**46.2 (45.1-53.4)**	47.1 (44.0-53.6)	**<0.001**	46.9 (39.0-52.9)	47.8 (42.9-57.2)	48.1 (44.2-55.6)	0.078
		**PPA lower**	33.3 (30.0-40.4)	35.6 (31.1-39.0)	**36.9 (31.5-40.0)**	**0.002**	36.3 (29.4-41.1)	35.8 (31.1-44.6)	36.9 (32.1-44.1)	0.316
	**high trypsin**	**entire**	37.2 (34.4-40.6)	36.9 (35.9-42.3)	36.2 (34.6-40.8)	0.316	40.4 (35.6-45.7)	39.2 (35.3-45.4)	**37.3 (34.4-42.5)**	**<0.001**
		**SPA**	37.0 (33.6-40.2)	36.5 (35.2-41.6)	35.9 (33.3-38.5)	0.078	40.9 (35.4-45.5)	38.6 (34.6-44.1)	**35.7 (33.7-39.9)**	**<0.001**
		**SPA upper**	44.6 (39.4-49.1)	45.5 (39.2-51.9)	42.5 (39.6-45.3)	0.066	47.4 (44.6-56.1)	46.2 (42.6-51.4)	**42.6 (39.9-44.7)**	**<0.001**
		**SPA lower**	30.8 (25.8-33.9)	31.3 (25.9-35.5)	31.8 (27.7-35.6)	0.078	32.9 (26.0-39.7)	34.6 (26.3-40.3)	34.6 (27.0-36.9)	0.710
		**PPA**	37.4 (35.6-41.5)	38.3 (36.9-44.1)	38.0 (36.6-45.3)	0.187	39.3 (36.5-48.7)	39.8 (38.2-49.3)	40.4 (38.1-49.2)	1.000
		**PPA upper**	44.1 (40.6-48.5)	46.9 (40.1-52.5)	46.9 (41.5-52.5)	0.316	46.5 (42.1-56.5)	47.2 (43.5-58.1)	47.3 (42.4-58.0)	0.710
		**PPA lower**	35.2 (26.6-36.8)	35.5 (26.2-37.9)	36.9 (28.1-38.7)	0.316	35.0 (27.3-41.3)	38.5 (27.3-40.9)	39.0 (29.1-41.6)	0.436
	**controls**	**entire**	44.3 (38.0-46.4)	44.0 (40.1-46.5)	42.1 (38.3-46.3)	0.107	35.6 (34.6-41.0)	35.9 (34.8-39.0)	34.8 (34.0-37.6)	0.328
		**SPA**	43.8 (36.9-45.3)	42.5 (38.9-46.7)	40.8 (36.8-46.2)	0.057	35.0 (34.2-40.0)	35.1 (34.5-37.7)	33.9 (33.3-35.8)	0.107
		**SPA upper**	47.8 (46.6-52.8)	48.3 (45.7-54.9)	**45.6 (42.1-51.7)**	**<0.001**	45.1 (38.8-46.1)	40.7 (39.6-46.6)	39.4 (37.7-44.0)	0.016
		**SPA lower**	37.5 (35.8-42.8)	38.9 (33.0-39.3)	39.5 (34.6-40.0)	1.000	29.5 (26.7-36.8)	30.4 (26.2-34.7)	28.5 (25.9-33.9)	0.814
		**PPA**	45.2 (42.6-49.7)	45.5 (44.8-48.7)	44.8 (43.7-46.4)	0.328	38.8 (35.1-43.1)	40.1 (37.3-41.7)	38.1 (35.2-41.3)	0.814
		**PPA upper**	50.9 (44.0-52.5)	49.9 (49.1-54.4)	48.3 (46.4-51.1)	**0.010**	45.4 (39.7-45.5)	45.9 (41.8-48.6)	44.1 (41.8-47.6)	0.569
		**PPA lower**	40.6 (39.1-45.9)	43.1 (39.3-45.3)	41.5 (39.0-44.1)	0.569	35.5 (26.3-38.0)	32.4 (29.5-38.1)	33.2 (29.1-38.9)	0.971
**T2***	**low trypsin**	**entire**	32.0 (29.6-36.5)	**35.0 (32.4-39.8)**	35.2 (33.5-37.6)	**0.003**	35.0 (32.7-38.6)	36.4 (32.5-40.4)	35.9 (32.8-38.9)	0.018
		**SPA**	31.2 (28.5-37.0)	34.7 (31.5-40.1)	34.7 (32.7-37.1)	0.046	35.3 (31.1-38.0)	36.5 (32.0-39.2)	35.6 (31.4-38.1)	0.046
		**SPA upper**	39.4 (35.6-44.7)	42.3 (39.8-44.3)	41.0 (38.7-43.2)	0.222	44.3 (35.3-47.1)	44.4 (37.2-46.5)	43.2 (37.4-44.9)	0.187
		**SPA lower**	23.2 (21.5-30.0)	**26.3 (24.4-35.8)**	26.7 (25.7-31.3)	0.012	27.2 (23.1-31.1)	**29.0 (24.9-34.7)**	28.3 (25.0-33.6)	**<0.001**
		**PPA**	33.3 (30.7-37.1)	**36.6 (33.0-38.5)**	**37.1 (33.2-38.5)**	**<0.001**	35.8 (31.2-40.4)	**36.6 (33.7-42.7)**	37.0 (34.9-41.0)	**0.006**
		**PPA upper**	38.9 (34.5-42.8)	**41.6 (38.2-43.6)**	**41.8 (38.5-44.9)**	**0.001**	42.0 (35.0-47.2)	41.5 (37.7-48.7)	42.5 (38.8-47.3)	0.368
		**PPA lower**	26.9 (23.6-33.1)	**30.0 (26.4-34.2)**	**30.9 (25.7-35.4)**	**<0.001**	29.8 (24.7-33.2)	30.8 (26.8-36.6)	**32.0 (26.4-36.6)**	**0.006**
	**high trypsin**	**entire**	31.6 (29.7-34.0)	32.0 (30.9-36.9)	31.3 (30.1-35.0)	0.018	32.7 (30.6-39.8)	33.0 (30.6-40.2)	31.0 (29.9-36.7)	0.018
		**SPA**	31.8 (27.8-34.1)	31.5 (30.3-36.6)	30.7 (28.0-33.6)	0.135	33.5 (29.6-40.0)	33.2 (30.1-39.7)	30.5 (28.9-34.8)	0.026
		**SPA upper**	38.3 (33.0-44.0)	39.1 (33.6-45.0)	37.0 (33.5-39.8)	0.026	41.0 (36.3-50.9)	39.7 (36.3-46.9)	**37.5 (33.2-40.8)**	**0.006**
		**SPA lower**	23.4 (21.2-27.2)	26.2 (21.9-29.7)	25.3 (23.5-29.6)	0.046	26.3 (21.7-29.9)	29.9 (22.3-34.4)	25.9 (22.4-29.9)	0.135
		**PPA**	31.9 (29.6-34.8)	**33.1 (31.7-38.2)**	33.0 (31.7-38.3)	**0.006**	31.1 (29.4-41.9)	34.5 (31.8-42.1)	33.1 (29.8-41.3)	0.066
		**PPA upper**	38.1 (35.6-41.9)	41.3 (36.0-45.5)	40.7 (37.7-46.8)	0.066	38.5 (35.5-50.3)	40.6 (36.8-48.6)	39.1 (36.6-49.9)	0.601
		**PPA lower**	26.0 (21.1-30.1)	**31.0 (21.7-31.8)**	30.4 (22.0-33.4)	**0.006**	27.9 (20.3-32.3)	33.1 (21.5-36.4)	27.1 (21.2-34.2)	0.046
	**controls**	**entire**	35.1 (30.5-37.9)	35.5 (31.2-40.2)	34.2 (30.0-39.8)	0.069	30.6 (29.1-34.2)	30.7 (29.8-32.9)	29.6 (28.8-31.2)	0.154
		**SPA**	36.1 (30.9-37.2)	34.1 (31.4-39.5)	33.8 (30.3-39.3)	0.107	30.2 (29.5-33.6)	30.6 (29.8-32.0)	29.4 (28.8-29.9)	0.048
		**SPA upper**	39.8 (36.5-45.6)	41.0 (36.9-47.5)	38.5 (35.4-46.4)	0.031	38.4 (34.0-39.7)	34.5 (32.3-40.6)	**35.7 (30.4-38.0)**	**0.010**

		**SPA lower**	30.2 (22.6-32.5)	32.3 (21.9-33.1)	31.8 (21.3-33.0)	0.569	23.9 (21.8-30.4)	25.5 (21.7-30.6)	24.4 (21.0-29.0)	0.971
		**PPA**	34.9 (30.7-40.6)	39.1 (31.6-40.2)	36.4 (30.4-38.2)	0.107	33.3 (28.5-35.2)	34.5 (30.2-34.6)	32.0 (30.1-33.7)	0.685
		**PPA upper**	37.9 (35.9-44.4)	42.3 (40.9-45.9)	39.9 (37.5-43.4)	0.278	37.8 (34.1-39.5)	38.8 (36.1-42.7)	36.4 (34.0-40.1)	0.398
		PPA lower	31.2 (22.7-31.9)	35.2 (26.7-36.1)	32.9 (24.6-34.5)	0.685	25.8 (21.3-30.4)	26.2 (24.5-30.9)	26.1 (23.9-31.4)	0.685

Supplementary Figure S1 (online only)

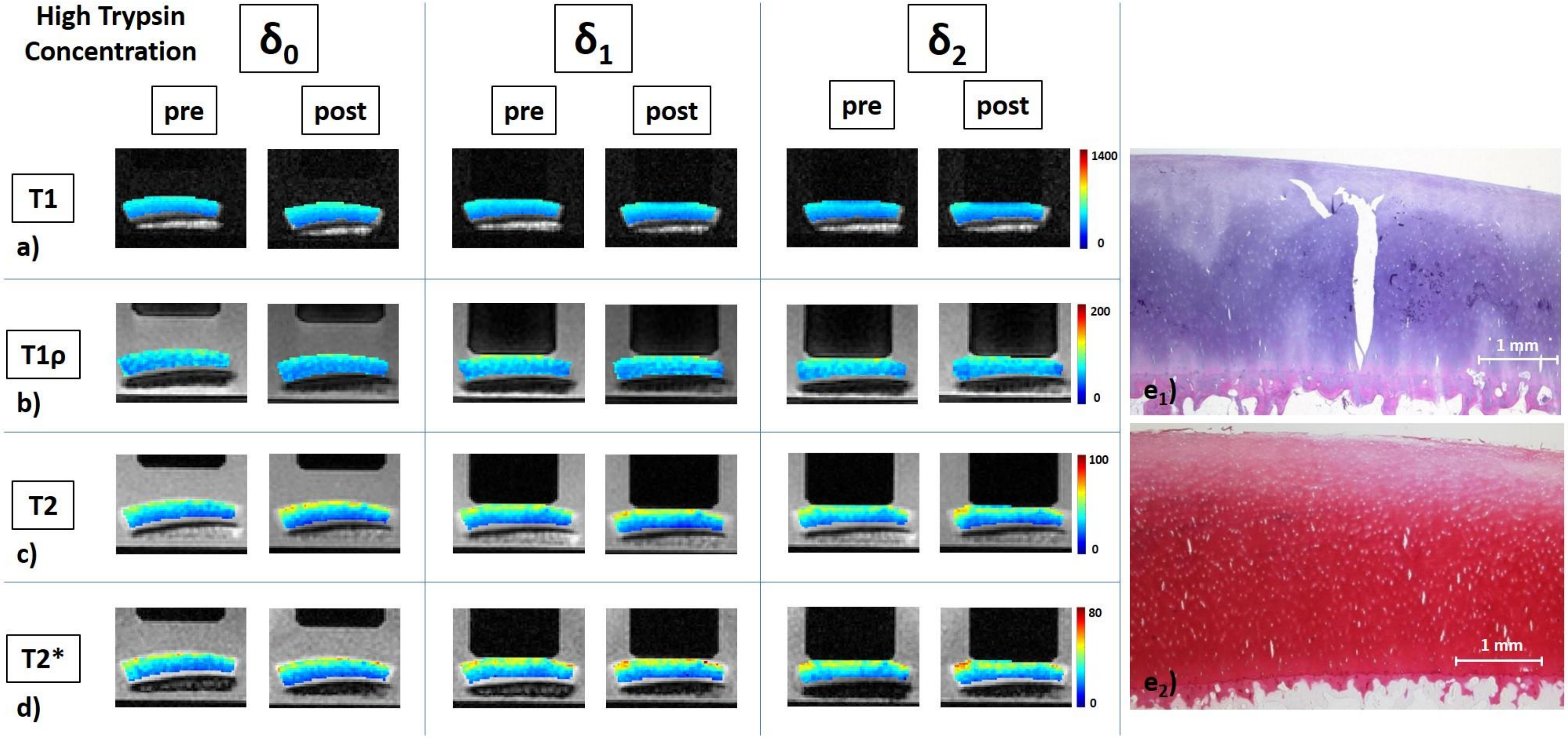

Supplementary Figure S2 (online only)

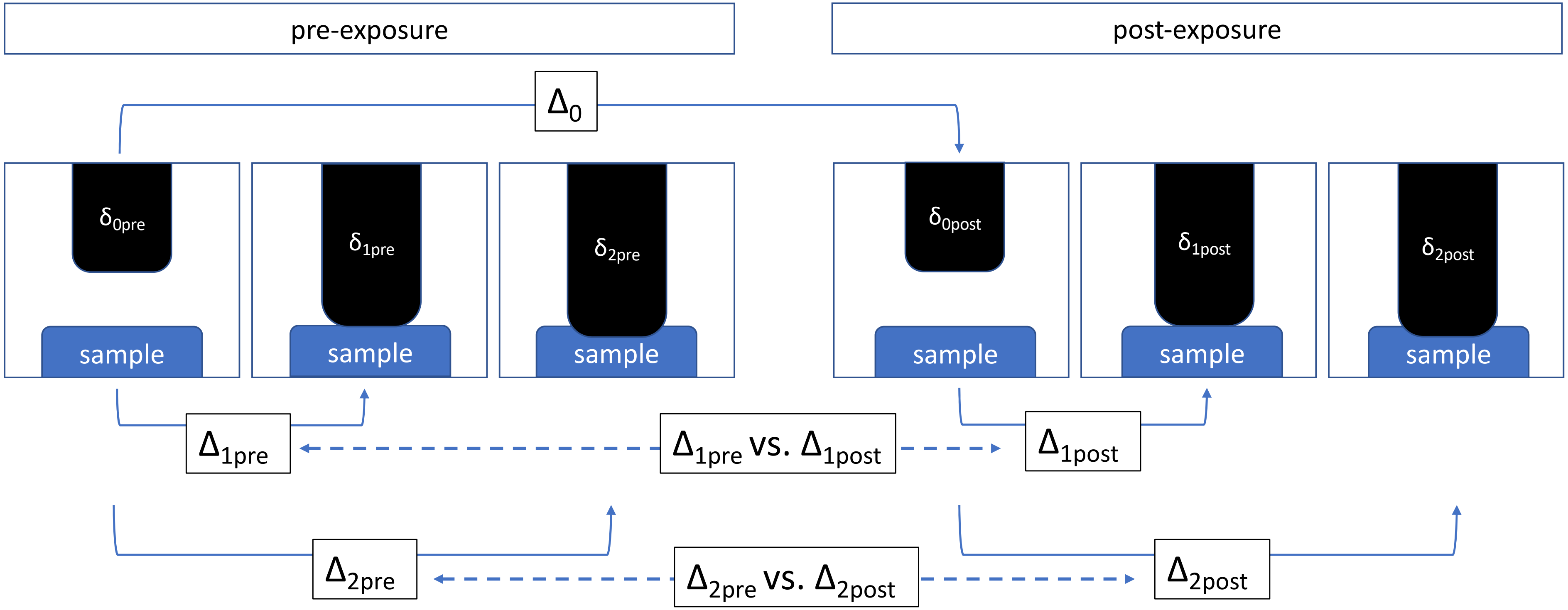

Supplement: Supplementary file 1 — Supplementary Information. [file 41598_2020_72208_MOESM1_ESM.pdf]
