# Supplementary material for: A serial multiparametric quantitative magnetic resonance imaging study to assess proteoglycan depletion of human articular cartilage and its effects on functionality
Source: Sci Rep. 2020 Sep 15;10:15106. doi: 10.1038/s41598-020-72208-y (PMC7492285; doi:10.1038/s41598-020-72208-y)
Supplement: Supplementary file 2 — Supplementary Legends. [file 41598_2020_72208_MOESM2_ESM.docx]

**Supplementary Material**

**Supplementary Table S1:** Mean cartilage sample height as a function of trypsin exposure and increasing loading intensity, i.e. unloaded (δ_0_) and loaded to 15.1 N (δ_1_) and 28.6 N (δ_2_), respectively. Sample height was determined on mid-sagittal PD-weighted images before (pre exposure) and after (post exposure) incubation and trypsin exposure. Controls were not treated. Data are means ± standard deviations and ordered as sample height [mm] (percentage change vs. δ_0_ [%]). Repeated measures ANOVA was used to assess loading-induced decreases in sample height that were significant (indicated in **bold type**). Pre- vs. post-exposure sample heights at δ_0_ were compared using paired Student's t-test and not significantly different; p-values were p=0.373 (†), p=0.343 (‡), and p=0.016 (§).

**Supplementary Table S2:** Pixel numbers in the distinct regions-of-interest as a function of loading intensity before (pre exposure) and after (post exposure) incubation and trypsin exposure. Data are means ± standard deviations. Regions-of-interest were defined as the entire cartilage sample (ECS), the sub-pistonal area (SPA), and the peri-pistonal area (PPA) as well as their upper (SPA_upper_, PPA_upper_) and lower subregions (SPA_lower_, PPA_lower_). For pixel numbers, loading-induced differences were assessed by repeated measures ANOVA. Significant differences are indicated in **bold type**.

**Supplementary Table S3:** Absolute T1, T1ρ, T2, and T2* values in the distinct regions-of-interest as a function of trypsin exposure and loading intensity. Dara are medians (interquartile ranges) [ms]. Regions-of-interest are defined in **Supplementary Table 2** and visualized in **Figure 2**. ROI-specific differences under loading were assessed using Friedman's test followed by Dunn's post-hoc test. Significant differences versus δ_0_ are indicated in **bold type**.

**Supplementary Figure S1:** Details of a high-concentration trypsin-treated cartilage sample, its response to loading, and corresponding histological sections.

Serial quantitative T1 (a), T1ρ (b), T2 (c), and T2* (d) maps as well as corresponding histological sections (e) of a representative sample before and after exposure to high concentration of trypsin at 1.0 mg/mL. Image details as in **Figure 3**.

**Supplementary Figure S2:** Schematic overview of the most important comparisons. δ_0_ refers to the absolute qMRI parameter values in the unloaded reference configuration, while δ_1_ and δ_2_ refer to the values under loading of 15.1 N (δ_1_) and 28.6 N (δ_2_). Relative changes at δ_1_ or δ_2_ versus δ_0_ are referred to as Δ_1_ or Δ_2_. Pre(-exposure) and post(-exposure) refer to the MRI measurements before (pre) and after (post) exposure to incubation with or without additional trypsin exposure.
